# Dietary intervention through bacterial-derived butyrate elicits anti-tumor activity and increases anti-PD-1 response

**DOI:** 10.1080/19490976.2026.2699457

**Published:** 2026-07-15

**Authors:** Myriam Benlaïfaoui, Corentin Richard, Sébastien Hunter, Eder Orlando Méndez-Salazar, Sylva Kourtian, Julie Malo, Diogjena Katerina Prifti, Mayra Ponce, Soumia Khalfi, Wiam Belkaïd, Meriem Messaoudene, Romain Boidot, Caroline Truntzer, François Girhinghelli, Catherine Lehoux-Duboix, Petronela Ancuta, Arielle Elkrief, Valérie Marcil, Bertrand Routy

**Affiliations:** a Centre de Recherche du Centre Hospitalier de Montréal (CRCHUM), Montréal, Québec, Canada; b Département de Microbiologie, infectiologie et Immunologie, Faculté de Médecine, Université de Montréal, Montreal, Québec, Canada; c Université Bourgogne Europe, Centre Georges-François Leclerc, Unicancer, Unité de Biologie Moléculaire, ICMUB UMR CNRS 6302, Dijon, France; d Université Bourgogne Europe, Centre Georges-François Leclerc, Unicancer, Cancer Biology Transfer Platform, INSERM, CTM UMR 1231, TIRECs Team, Dijon, France; e Département de Nutrition, Faculté de Médecine, Université de Montréal, Montreal, Québec, Canada; f Oncology Division, Department of Medicine, University of Montreal Hospital Centre (CHUM), Montreal, Quebec, Canada; g Centre de Recherche Azrieli du CHU Sainte-Justine, Montréal, Québec, Canada

**Keywords:** Dietary fiber, cancer, immunotherapy, NSCLC, gut microbiome, SCFA, butyrate

## Abstract

The gut microbiome is increasingly recognized as a key modulator of cancer immunotherapy efficacy. Given that diet is one of the most important determinants of the gut microbiome composition and function, nutritional strategies have emerged as promising tools to modulate anti-tumor immune responses. Here, we demonstrate that dietary supplementation with inulin reduces tumor growth and enhances **α**PD-1 efficacy in mice. These effects were associated with increased frequencies of intra-tumoral CD8⁺ and CD4⁺ T cells, particularly CCR9⁺CXCR3⁺ subsets, and enrichment of beneficial taxa such as *Akkermansia* and *Lachnospiraceae*, alongside elevated short-chain fatty acids (SCFA) levels. Among the SCFA, butyrate alone recapitulated the anti-tumor effect of inulin and had an additive effect when combined with **α**PD-1 therapy in a CD8⁺ T cell-dependent manner. Butyrate exerted its anti-tumor effects by transcriptional changes in CD8⁺ T cells involving activation of proliferation, trafficking, and metabolic pathways. In a cohort of 117 non-small cell lung cancer (NSCLC) patients amenable to immunotherapy, the median dietary fiber intake was lower than previously published studies but correlated with enrichment of *Faecalibacterium praunitzii* and metabolic pathways related to sucrose degradation and tryptophan biosynthesis. Collectively, our findings highlight the therapeutic potential of targeting diet-microbiome-immune system interactions to improve cancer immunotherapy outcomes.

## Introduction

Despite the success of immune checkpoint inhibitors (ICI) in treating patients with non-small cell lung cancer (NSCLC), most patients ultimately experience disease progression.^1^ ^,^ ^2^ Over the past decade, the composition of gut microbiota has been recognized as a major determinant of ICI activity, with specific bacteria such as *Akkermansia muciniphila* and members of the *Lachnospiraceae* family being positively associated with response to ICI.^3-5^ These findings led to the development of strategies such as the transfer of fecal microbiota from healthy donors, probiotics, or prebiotics in interventional trials.^6-9^ However, it is key to consider environmental factors, with dietary habits being one of the most important determinants of the gut microbiome composition, and a scalable and direct strategy to reshape the gut microbiota composition.^10^ Among many dietary interventions, fiber supplementation has shown feasibility in early-phase clinical trials.^11^ ^,^ ^12^ However, how fiber intake modulates the gut microbiome, and more importantly, the response to ICI in patients with NSCLC, remains unknown.

Dietary fibers are carbohydrate polymers classified based on their fermentability by the gut microbiome.^13^ In pre-clinical models, supplementation of fermentable fiber, such as an inulin diet, has been associated with increased Th1 activity and enhanced anti-tumor activity.^14^ ^,^ ^15^ In patients with melanoma treated with ICI, studies have linked sufficient dietary fiber intake (>20 g/d) or high omega-3 fatty acid intake (>250 mg/d) to enhanced clinical responses.^16^ ^,^ ^17^ Similar outcomes were also observed in Mediterranean diet, known to be rich in fiber, polyphenols, and unsaturated fatty acids, and has been associated with positive objective response rates (ORR) in 91 melanoma patients treated with ICI.^18^ In particular, high fiber and Mediterranean diets have been linked to increasing the production of short-chain fatty acids (SCFA) such as butyrate, propionate, and acetate, which are generated through bacterial fermentation of complex carbohydrates.^19^ SCFA exert context-dependent functions on the immune response. In colorectal cancer, butyrate-producing bacteria help maintain gut homeostasis by reducing local inflammation and expanding FoxP3^+^ regulatory CD4^+^ T cells via G-protein-coupled receptor (GPCR) activation.^20^ However, in the context of cancer immunotherapy, SCFA, particularly butyrate, can enhance CD8^+^ T cell function.^21^ ^,^ ^22^ Recent studies have demonstrated that microbiota-derived butyrate and pentanoate promote effector molecule production in CD8^+^ T cells and chimeric antigen receptor (CAR) T cells via histone deacetylase (HDAC) inhibition, resulting in enhanced tumor control in mouse models.^23^ On the other hand, elevated systemic butyrate levels in the blood were associated with **α**CTLA-4 resistance, highlighting the complexity and dual role of SCFA in oncology.^24^

Despite this growing body of literature linking the role of diet on bacteria-derived metabolites and oncology, the impact of diet on microbiome composition and clinical outcomes in patients with NSCLC treated with ICI remains poorly understood. The American Society of Clinical Oncology guidelines report that there is no evidence to currently recommend any diets for patients on active treatment.^25^ Moreover, the therapeutic potential of microbiome-derived SCFA in enhancing ICI efficacy has not been fully investigated.

In this study, inulin supplementation in mice led to a favorable remodeling of the gut microbiome composition, accompanied by increased SCFA fecal levels. Moreover, oral butyrate supplementation had an additive effect with **α**PD-1 through the activation of gut-homing receptors and transcriptomic reprogramming of tumor-infiltrating CD8^+^ T cells. In a cohort of NSCLC, we showed that despite a low median fiber intake, fiber correlated with enhanced relative abundance of beneficial *Faecalibacterium prausnitzii*.

## Materials and methods

### Murine and in vitro studies

#### Mice

Mice experiments were conducted in the CRCHUM animal facility after ethical approval from the *Comité institutionnel de protection des animaux* (CIPA, ethics number: C23046BR). Animals consisted of female C57BL/6 mice, aged 6 weeks, and were purchased from Charles River. After arrival, mice were kept in pathogen-free (SPF) conditions. All animal studies were approved and conducted in compliance with Canadian laws and regulations. At the end of each experiment, mice were euthanized under deep anesthesia induced with 3–4% isoflurane administered in an induction chamber with oxygen delivered at 1 L/min. To ensure that anesthesia has worked, the absence of reflex was verified before performing cervical dislocation.

#### Cell lines

The murine fibrosarcoma cell line MCA-205 and Ovalbumin-expressing B16 melanoma cells were cultured in Roswell Park Memorial Institute (RPMI; Gibco) 1640 medium, while the E0771 murine mammary carcinoma cells were cultured in Dulbecco's Modified Eagle's Medium (DMEM; Gibco). All media were supplemented with 10% fetal bovine serum (FBS), 1% penicillin/streptomycin, and 1% L-glutamine. Cultures were maintained at 37 °C in a 5% CO_2_ atmosphere.

#### Subcutaneous tumor injection

As previously described, tumor cells were injected subcutaneously into the right flank of mice, using either 0.5 × 10^6^ B16-OVA cells, 0.8 × 10^6^ MCA-205 cells, or E0771 cells.^26^ Tumor length (L) and width (W) were measured using a caliper, and tumor volume was calculated as *V* = 4/3 π*(length/2)*(width/2)^2^.

When the tumor surface reached 30–35 mm^2^, mice were randomized into treatment groups based on tumor size to ensure balanced baseline tumor burden across groups. Mice were then treated intraperitoneally with either an anti–PD-1 antibody (100 μg/mouse; clone RMP1, BioXcell) or an isotype control (clone 2A3, BioXcell). Mice received four injections of treatment (anti–PD-1 or isotype control) at 3-d intervals for the B16-OVA and MCA-205 models, and five injections for the E0771 model. Tumor measurements and fecal samples were collected at each treatment time point. Investigators were not blinded to group allocation during treatment administration, tumor measurements, or sample collection. No predefined exclusion criteria were applied, except veterinary-mandated euthanasia (e.g., ulceration/necrosis). In such cases, animals were included in longitudinal analyses up to their last available measurement; endpoint analyses included only mice with available measurements at sacrifice.

#### Fiber diet

Mice received either the standard chow diet from the animal facility (Teklad Global 18% Protein Rodent Diet), a low fermentable fiber diet containing 10% Cellulose (TD190723) or a high fermentable fiber diet containing 10% Inulin (TD190651). All diets were irradiated and administered *ad libitum* under sterile conditions. Diet administration started 2 weeks before tumor injection and continued until the mice sacrifice. The fiber source and macronutrient composition of each diet are summarized in Supplementary Table 1.

#### SCFA administration

Sodium acetate (#CAT: S2889; Sigma-Aldrich), sodium butyrate (#CAT: 303410; Sigma-Aldrich), and sodium propionate (#CAT: P1880; Sigma-Aldrich) were reconstituted in sterile water at a concentration of 100 mM. The solutions were given to the mice in a drinking bottle and were changed every 3 d.

#### Antibiotics treatment

Mice received a 3-d antibiotic cocktail (ABX) containing ampicillin (1 mg/mL; Sigma-Aldrich), streptomycin (5 mg/mL; Sigma-Aldrich), and colistin (1 mg/mL; Sigma-Aldrich), administered in sterile drinking water prior to FMT to deplete the endogenous recipient microbiota. The efficiency of microbiota depletion was assessed before FMT by collecting fecal pellets from antibiotic-treated mice, resuspending them in sterile NaCl, and plating the suspension on Columbia agar plates supplemented with 5% sheep blood (COS; BD BBL). Plates were incubated for 24 h at 37 °C under both aerobic and anaerobic conditions. Absence of detectable bacterial growth was used to confirm efficient depletion of bacteria prior to FMT, which was performed within 12 h after antibiotic discontinuation.

#### Fecal microbiota transplantation (FMT)

Patients' fecal material was thawed and resuspended in NaCl. Two hundred μL of the suspension was then administered to the antibiotic (ABX) pre-treated mice via oral gavage. Additionally, 100 μL of the suspension was applied to the fur of each animal. Two weeks after FMT, tumor cells were injected subcutaneously, and mice were treated with **α**PD-1 or isotype controls as mentioned above.

#### SCFA stool quantification

Stool samples were analyzed by Liquid Chromatography with tandem Mass Spectrometry (LC-MS/MS) after derivatization using a previously published method.^27^ An external calibration curve comprising a mix of all the analytes was employed, and 2,2-dimethylbutyric acid was used as an internal standard.

#### Dissociation and preparation of cell suspensions

Tumors and spleens were harvested from mice at sacrifice for flow cytometry analysis. Tumors were minced and digested in a dissociation medium containing RPMI, Liberase (Roche) at 25 mg/mL, and DNase1 (Roche) at 150 U/mL for 30 minutes at 37 °C. The mixture was then passed through a 70μm cell strainer. Following filtration and washing with RPMI supplemented with 10% FBS, lymphocyte cells were counted after dead cell exclusion using trypan blue staining. Cells were resuspended in PBS1X + 10% FBS at a concentration of 0.2 × 10^6^ cells/200 μL for flow cytometry. For spleens, cells were collected after tissue homogenization through a 100 μm filter using a syringe plunger, and red blood cells were lysed with Ammonium–Chloride–Potassium (ACK) lysis buffer. Cells were washed, counted, and prepared for flow cytometry staining following the same steps as for tumors.

#### Flow cytometry

Cells obtained after dissociation were pre-incubated with purified anti-mouse CD16/CD32 (clone 2.4G2, Tonbo Bioscience, Cat#70-0161-M001) for 30 minutes at 4 °C before surface staining. Dead cells were excluded using the Live/Dead Aqua cell staining kit (Invitrogen, CAT#L34957). Anti-mouse CD4 (clone GK1.5, FITC, BioLegend, Cat#100406); CD8 (clone 53-6.7, PE, eBioscience, Cat#12-0081-83); CD3 (clone 145-2C11, APC, BD Biosciences, Cat#553066); CD45 (clone 30-F11, PerCP-Cy5.5, BD); Foxp3 (clone FJK-16s, PE-Cy7, eBioscience); CXCR3 (clone FAB1685P, APC, R&D); CCR9 (clone W-1.2, PE, BioLegend); PD-1 (clone 29F.1A12, BV421, BioLegend); PD-L1 (clone MIH5, FITC, eBioscience) were used to stain the cells. For intracellular staining, the Foxp3 staining kit (eBioscience, CAT#00-5523-00) was used. Stained samples were acquired on a FACSSymphony A3 flow cytometer (BD Bioscience), and data analysis was performed using FlowJo software v10.8.1 (Tree Star, Ashland, OR, USA).

#### Tissue microarray (TMA) construction

TMA blocks from MCA-205 tumors of standard or inulin isotype or **α**PD-1 were built using the TMArrayer (Pathology Devices, Inc.) as previously described.^28^ TMA sectioning was done with a Sakura Microtome at 4 μm thickness. A total of three cores from each tumor tissue (0.6mm) were transferred to a recipient TMA block in a randomized manner.

#### COMET multiplex immunofluorescence

Following standard deparaffinization, 4 µm-thick sections of the FFPE tissue blocks were treated using the Discovery Ultra Ventana automated stainer (Ventana Medical Systems, Roche, Rotkreuz, Switzerland). Antigen retrieval was conducted with Cell Conditioning #1 solution (Ventana Medical System Inc., Tris-EDTA buffer, pH 7.8) for 60 minutes at 95 °C. A 20-plex protocol template was created using the COMET Control Software, after which all reagents were loaded onto the instrument to run the fully automated sequential immunofluorescence (seqIF) workflow, as previously described.^29^ Nuclear staining was achieved with DAPI (Thermo Scientific, cat. 62248, diluted 1:1000) either through a 2-minute dynamic incubation or by adding DAPI directly to the secondary antibody mixtures. During each staining round, primary antibody mixes were incubated dynamically for 2–8 minutes, depending on the antibodies, while secondary antibody solutions and DAPI mixtures were incubated for 2 minutes. Primary antibody cocktails; FoxP3 (Thermo Scientific, 14-5773-82, diluted 1:50), CD4 (Thermo Scientific, 14-9766-82, diluted 1:100), CD8 (Thermo Scientific, D4W2Z, diluted 1:200) were prepared in Multistaining Buffer (BU06, Lunaphore). For every imaging cycle, exposure times were set to 25 ms for DAPI, 250 ms for TRITC, and 400 ms for Cy5. Each cycle included a 2-minute elution step using Elution Buffer (BU07-L, Lunaphore), followed by a 30-second quenching step with Quenching Buffer (BU08-L, Lunaphore). Imaging was carried out in Imaging Buffer (BU09, Lunaphore). Secondary antibody mixtures consisted of either Alexa Fluor Plus 647 goat anti-rabbit (Thermo Scientific, A32733, diluted 1:200) paired with Alexa Fluor Plus 555 goat anti-mouse (Thermo Scientific, A32727, diluted 1:100), or Alexa Fluor Plus 647 goat anti-rabbit (Thermo Scientific, A32733, diluted 1:200) combined with Alexa Fluor Plus 555 goat anti-rat (Thermo Scientific, A48263, diluted 1:100) or Alexa Fluor Plus 647 goat anti-rat (Thermo Scientific, A48265, diluted 1:200). After the staining workflow was completed, the COMET Control software produced a raw OME-TIFF file for subsequent data processing.

#### 16S rRNA gene sequencing

Fecal samples were collected from mice under sterile conditions and stored at −80 °C until processing. DNA was extracted and purified using the ZymoBIOMICS DNA Kits (Zymo Research), following the manufacturer's instructions. The V3-V4 region of the 16S rRNA gene was amplified using primers Bakt_341F (5′-CCTACGGGNGGCWGCAG-3′) and Bakt_805R (5′-GACTACHVGGGTATCTAATCC-3′). Differential abundance analysis of microbial taxa was performed using MaAsLin2 in R (v4.3.1). Relative abundances were transformed using the centered log-ratio (CLR) method. Taxa were considered significantly differentially abundant if they showed an absolute log₂ fold change > 1 and a false discovery rate (FDR) < 0.05, with *p*-values adjusted by the Benjamini–Hochberg procedure. Results were visualized using the ggplot2 package in R. Observed, Shannon, and Inverse Simpson were calculated to assess the alpha diversity of each sample. Beta diversity was assessed using Bray–Curtis dissimilarity and visualized using PCoA or NMDS, as specified in the corresponding figure legends. Group differences in community composition were tested using PERMANOVA.

#### RNA-sequencing

Total RNA was isolated using the Monarch Total Miniprep kit (New England Biolabs). For each sample, 50 ng of total RNA underwent ribosomal RNA depletion using the RiboCop rRNA Depletion kit (Lexogen), following the manufacturer's guidelines. Next, sequencing libraries were prepared with the SMARTer Ultra Low Input RNA for Illumina (Takara). Libraries were sequenced in paired-end (2 × 75 bp) on the AVITI sequencing platform (Element Biosciences) with a depth of 25 million reads. Bases2fastq software (Element Biosciences, v2.2.0) was used to demultiplex the raw data. Transcripts per kilobase million (TPM) were quantified using Kallisto software and GRCm37 assembly Ensembl.^30^ Gene-level count and transcript matrices were estimated with the DESeq2 R package. Genes with fewer than five total reads across all samples were excluded before differential expression analysis. Differential gene expression was assessed in R using the DESeq2 package. Differentially expressed genes (DEGs) were defined as those with an adjusted *p*-value < 0.05. For visualization of overlaps (UpSet plots and Venn diagrams), an additional fold-change threshold was applied (FC > 1.25 for upregulated genes and FC < 0.8 for downregulated genes), as indicated in the corresponding figure legends. Gene set enrichment analysis (GSEA) was performed on the resulting differential genes using hallmarks of cancer gene sets from the Broad Institute and the fgsea R package.^31^ To characterize the immune landscape from bulk RNA-seq data, ImmuCellAI software estimated the relative abundance of 24 immune cells comprising 18 T-cell subtypes and 6 other immune cells: B cell, NK cell, Monocyte cell, Macrophage cell, Neutrophil cell, and DC cell.^32^

#### CD4 and CD8 T cell isolation

Human CD4^+^ and CD8^+^ T cells were isolated from healthy subject PBMC by negative selection using magnetic beads following the manufacturer's instructions (magnetic-activated cell sorting (MACS), Miltenyi Cat# 130-096-533 and Cat# 130-096-495). The untouched CD4^+^ and CD8^+^ T cells were collected from the flow-through fraction. Cell purity was routinely assessed by CD3 (clone UCHT1, Pacific Blue, BD Biosciences, Cat#558117), CD16 (clone 3G8, PE-Cy7, BD Biosciences, Cat#557744), CD4 (clone RPA-T4, Alexa Fluor 700, BD Biosciences, Cat#557922), and CD8 (clone RPA-T8, FITC, BD Biosciences, Cat#555366) antibodies and was consistently greater than 95%. After isolation, cells were counted to achieve a concentration of 2 million/mL in an RPMI1640 medium (Gibco) containing 10% FBS and 1% P/S.

#### SCFA and AhR ligands treatment

The previously CD4^+^ or CD8^+^ purified cells were placed in a 48-well plate (1 millions/500 μL) in the presence of immobilized CD3 and soluble CD28 antibodies (1 μg/mL, BD Pharmingen). For SCFA treatments, cells were cultured in RPMI1640 medium (Gibco) containing 10% FBS and 1% P/S and in the presence or the absence of sodium butyrate (0.5 or 1 mM) or sodium acetate (10 mM). For the aryl hydrocarbon receptor (AhR) treatments, the antagonist CH-223191 (10 μM, Sigma) and the agonist FICZ (100 nM, Sigma) were added in their respective wells. After 2 d of co-culture, cells were harvested and washed, then stained with Fixable Viability Stain 575 V (BD Biosciences, Cat#565694), CD3 (clone UCHT1, BUV805, BD Biosciences, Cat#612895), CD4 (clone RPA-T4, BUV496, BD Biosciences, Cat#564651), CCR9 (clone C9Mab-1, APC, BD Biosciences, Cat#567976), and Lymphocyte Peyer's Patch Adhesion Molecule-1 (LPAM-1)/Integrin **α**4β7 (clone ACT-1, Alexa Fluor 647, BD Biosciences, Cat#571600).

### Human studies

#### Cohort description

The non-small cell lung cancer cohort comprised 117 individuals, primarily diagnosed with lung adenocarcinoma, and squamous cell carcinoma was included in the study. Clinical data, including line of immunotherapy, specific anti-PD-1 agents administered, and use of antibiotics, were extracted from medical records. Tumor response was evaluated on CT scans using Response Evaluation Criteria in Solid Tumors (RECIST) version 1.1. Progression-free survival (PFS) and overall survival (OS) were calculated from the start of anti-PD-1 therapy to the date of documented progression or death. The study was approved by the institutional ethics committee (CHUM Research Center: MP-02-2018-7132) and conducted in accordance with the Declaration of Helsinki and the guidelines of Good Clinical Practice. All patients provided written, informed, and signed consent prior to study participation.

#### Food frequency questionnaire

Patients' dietary intake was determined by using a modified version of a previously published short food frequency questionnaire (FFQ).^33^ The FFQ consisted of 47 items, and participants were instructed to choose a frequency option for each food item, ranging from never to a maximum of 5 times per week. Portion sizes for each food item were specified to facilitate accurate estimation of dietary intake. Nutrient intake assessment was conducted using a calculation tool that had been validated previously.^34^

#### Metagenomics sequencing

Stool samples were self-collected by patients at home following the International Human Microbiome Standards (IHMS) SOP 003. Immediately after collection, samples were placed at 4 °C in a collection container stored in a sealed bucket with an anaerobic bag, and subsequently transported by the patient to the hospital. Upon reception at the research site, samples were stored at −80 °C. Because collection was performed at home, the exact time from collection to −80 °C storage could not be fully standardized; however, in most cases, samples remained at 4 °C for an estimated 24–48 h prior to −80 °C storage. Shotgun metagenomics sequencing was performed to assess fecal microbial composition, as previously described.^35^ Briefly, after DNA extraction, sequencing libraries were prepared using Illumina® DNA Prep (M) Tagmentation kit (Illumina). Samples were sequenced using the NovaSeq 6000 S4 platform (Illumina), generating 150 bp end-paired reads. The Segata Lab pipeline (https://github.com/SegataLab/preprocessing) was used for quality control and processing. Microbial taxonomic profiling and quantification were conducted using MetaPhlAn 4.

#### Metagenomics analysis

Statistical analyses and visualizations were performed in R (4.4.2) as previously described.^26^ The phyloseq package (v.1.50.0) was used for taxonomic assignments. Alpha-diversity (richness, Shannon) was evaluated using the vegan package (v.2.7.1). Non-parametric tests (*p* < 0.05 considered significant) were used to calculate differences between groups. For the beta-diversity, Bray–Curtis dissimilarity was used and visualized with non-metric multidimensional scaling (NMDS) and Principal Coordinates Analysis (PCoA). Group differences were tested using PERMANOVA. Linear discriminant analysis (LEfSe) was performed using the package yingtools2 (v.0.0.1.184), retaining features with LDA score >2 and *p* < 0.05.

#### MetaCyc

Pathway analysis was performed on shotgun metagenomic sequencing data. Samples were processed using the HUMAnN3 pipeline (v3.8). MetaCyc pathway abundances were quantified, and stratified pathway profiles were used for downstream analyses. Pathways with zero total abundance across all samples were filtered out.^36^

### Statistical analysis

#### Experimental data analyses

Experimental data were analyzed using GraphPad Prism (v10.5.0, GraphPad Software, San Diego, CA, USA). For normally distributed data, statistical analyses were conducted using Student's t-test or one-way ANOVA. Non-parametric data were analyzed using Mann–Whitney *U*, Kruskal–Wallis tests, or Friedman tests, followed by Dunn's multiple comparisons test. Results are expressed as mean ± SEM. Statistical significance was defined by *p* < 0.05, and *p* > 0.05 was considered non-significant.

#### Clinical data analyses

Dietary intake and survival correlation was assessed using Spearman's rank correlation, and significance was determined with a two-tailed test, where a *p*-value < 0.05 was considered statistically significant. Statistical analyses were conducted using GraphPad Prism (v10.5.0, GraphPad Software, San Diego, CA, USA).

Multivariable survival analyses were performed using Cox proportional hazards regression models to estimate hazard ratios (HRs) and 95% confidence intervals (CIs) for dietary fiber intake while adjusting for clinical covariates (sex, smoking status, ECOG performance status, PD-L1 category, stage, and anti–PD-1 regimen [anti–PD-1 alone vs. combination]). Models were fitted using complete-case analysis due to missing covariate data (OS: *n* = 105; PFS: *n* = 106). Statistical significance was defined as a two-sided *p* < 0.05.

## Results

### High fermentable fiber diet enhances anti-tumor response and ICI efficacy in association with modulation of the gut microbiome composition and SCFA production in mice

**Figure 1.**
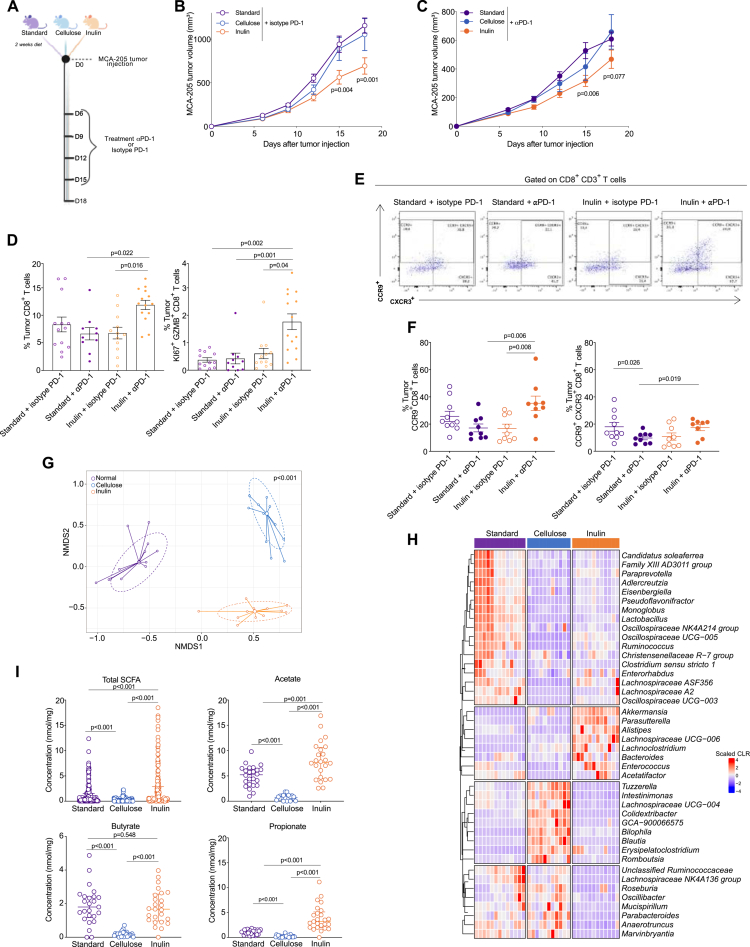
High Fermentable fiber diet modulates the gut microbiome composition to enhance anti-tumor response and ICI efficacy in mice. (A) Schematic representation of the experimental design of the dietary supplementation in mice. Standard (purple), cellulose (blue), and inulin (orange) diets were administered for two weeks before MCA-205 tumor injection and maintained until the end of the experiment. Mice in each group were further subdivided into **α**PD-1 or isotype control treatment groups. Tumor growth kinetics of subcutaneous MCA-205 tumor in mice fed a standard (isotype, *n* = 20; **α**PD-1, *n* = 20), cellulose (isotype, *n* = 15; **α**PD-1, *n* = 10), or inulin (isotype, *n* = 18; **α**PD-1, *n* = 14) diet, under isotype control (B) or **α**PD-1 (C) treatment. Results are shown from combined data from independent experiments; *n* refers to the number of mice analyzed per group. A mixed-effect analysis was conducted with time as a repeated measure. Treatment, including diet and iso/PD-1 treatment, was modeled as fixed effects, along with interaction terms to evaluate if growth curves separated over time. This analysis showed a significant effect of time (*p* < 0.0001) and significant time × treatment (*p* < 0.0001), indicating that tumor growth kinetics differed across diets and according to treatment over time. The main effect of treatment alone was not significant in this model (Treatment: *p* = 0.56). Selected cross-sectional comparisons (Wilcoxon tests), only between the standard diet (isotype or **α**PD-1) and inulin (isotype or **α**PD-1) at day 15 and day 18, are reported in the figure. (D) Multiplex analysis of tumor sections from standard and inulin diet groups (isotype or **α**PD-1) representing the frequency of CD8^+^ T cells and KI67^+^ GZMB^+^ CD8^+^ T cells. Each dot represents one individual tumor. Data from different biological and technical replicates were included in the analysis. (E) Dot plot representation of CCR9 and CXCR3 frequency gated on CD8⁺ CD3⁺ T cells isolated from MCA-205 tumors of mice fed a standard or inulin-enriched diet and treated with either isotype control or **α**PD-1 antibody. (F) Percentages of CCR9^+^ CD8^+^ T cells and CXCR3^+^ CCR9^+^ CD8^+^ T cells expression on tumor-infiltrating CD8^+^ CD3^+^ lymphocytes from MCA-205 mice fed a standard or inulin-enriched diet and treated with isotype control (respectively, *n* = 10; *n* = 9) or anti–PD-1 antibody (respectively, *n* = 9; *n* = 8). Results are shown from combined data from two independent experiments; *n* refers to the number of mice analyzed per group. (G) Non-metric multidimensional scaling (NMDS) plot based on Bray-Curtis dissimilarities derived from log-transformed bacterial community composition (V3-V4 16S rRNA gene data) from MCA-205 experiment. Data are illustrated as follows: mice fed with a standard diet in purple (*n* = 13); cellulose in blue (*n* = 11); inulin in orange (*n* = 12). (H) Heatmap representation of the relative abundance of dominant bacterial taxa in fecal samples from mice fed a standard (purple), cellulose (blue), or inulin-enriched (orange) diet. Each column represents one mouse. Values are CLR-transformed (centered log-ratio) and scaled as z-scores per taxon. (I) Fecal concentrations of total SCFA, acetate, butyrate, and propionate in the MCA-205 mouse experiment across dietary groups. For acetate, butyrate, and propionate, *n* refers to the number of mice with available quantification for each metabolite (acetate: standard *n* = 25, cellulose *n* = 23, inulin *n* = 24; butyrate: standard *n* = 25, cellulose *n* = 25, inulin *n* = 24; propionate: standard *n* = 25, cellulose *n* = 22, inulin *n* = 24). For total SCFA, *n* refers to the total number of individual metabolite measurements contributing to the calculated sum of SCFA analyzed (sum of acetate, butyrate, propionate, isobutyrate, isovalerate, succinate, lactate, and valerate), rather than the number of mice (standard *n* = 193, cellulose *n* = 184, inulin *n* = 182). Results are shown as mean ± SEM. Statistical significance was assessed using the Mann–Whitney *U* test.

Initially, we assessed the impact of diets containing fermentable and non-fermentable fibers on anti-tumor activity and their influence on the microbiome composition. Three distinct diets were tested in C57BL/6 female mice: a standard diet (containing 14.7% of a mix of cellulose, hemicellulose, and lignin), a low-fermentable fiber diet (containing 10% cellulose), and a high fermentable fiber diet (containing 10% inulin), whose macronutrient compositions are detailed in Supplementary Table 1. After two weeks of diet exposure, mice were subcutaneously injected with MCA-205 tumor cells and treated with ⍺PD-1 or an isotype control (Figure 1A). Longitudinal tumor growth analysis revealed that inulin administration significantly reduced tumor volume over time compared to the standard and cellulose diet groups, corroborating previously published studies in which fermentable fiber exerted a potent anti-tumor effect^14-16^ (Figure 1B). Consistently, tumor weight at sacrifice was significantly lower in inulin-supplemented mice compared to both the standard and cellulose diet groups (Supplementary Figure 1A). In addition, inulin significantly enhanced ⍺PD-1 activity compared to the two other diets (Figure 1C). Body weight remained comparable across dietary groups throughout the experiment (Supplementary Figure 1B). Although the inulin + isotype control group showed the lowest baseline body weight, its growth kinetics followed a trajectory similar to that of the other groups, suggesting no diet-related effect or palatability.

Using a multiplex immunofluorescence approach, we observed a significantly increased frequency of intratumoral FoxP3^-^ CD4^+^ T cells and CD8^+^ T cells in inulin + ⍺PD-1 mice compared to the standard diet group (Supplementary Figure 1C, Figure 1D). This increase was accompanied by a higher frequency of proliferating Ki67^+^ and cytotoxic Granzyme B^+^ CD8^+^ T cells, as well as an increase in the ratio of CD8^+^ T cells and FoxP3^+^ CD4^+^ T cells in inulin + ⍺PD-1 mice (Figure 1D, Supplementary Figure 1C). We confirmed these results using flow cytometry, which revealed an increase in tumor-infiltrating CD8^+^ T cells and reduced regulatory T cells in inulin + ⍺PD-1 mice (Supplementary Figure 1D). Flow cytometric analysis also revealed that inulin supplementation in combination with ⍺PD-1 led to a significant increase in the frequency of intra-tumoral CCR9⁺ CD4⁺ and CD8⁺ T cells (Figure 1E, F; Supplementary Figure 1E). Furthermore, a subset of these cells co-expressing CCR9 and the Th1-associated chemokine receptor CXCR3 was also enriched in tumors and spleens of inulin + ⍺PD-1–treated mice compared with the standard diet group (Figure 1F; Supplementary Figure 1F).

To explore the impact of these diets on microbiome composition, 16S rRNA sequencing was performed on murine fecal samples. Standard and cellulose diets were associated with the highest alpha diversity, whereas inulin may have limited its effect on specific fermentative taxa (Supplementary Figure 1G). Each diet independently altered the global microbiome composition as reflected by the change in beta diversity (*p* < 0.001) (Figure 1G). These microbiome shifts were independent of ⍺PD-1 treatment, as evidenced by the absence of clustering according to treatment groups (Supplementary Figure 1H). At the taxonomic level, heatmap and relative abundance representations analyses revealed that *Akkermansia, Alistipes,* and *Lachnospiraceae* UCG 006 were significantly enriched in the inulin group compared to the two other diets. Conversely, *Anaerotruncus* was increased in mice on the cellulose diet (Figure 1H; Supplementary Figure 1I).

To further explore the relationship between the microbiome and fiber fermentation, we quantified SCFA levels in the fecal samples of dietary-supplemented mice using liquid chromatography-tandem mass spectrometry (LC-MS/MS). Total SCFA levels, as well as acetate, butyrate, and propionate levels, were significantly increased in the inulin group compared to cellulose (Figure 1I). Conversely, lactic acid was the only SCFA significantly increased in the standard diet compared to inulin (Supplementary Figure 1J). Taken together, these results demonstrate that inulin supplementation is associated with beneficial shifts in microbiome composition and increased SCFA production, concurrent with an improved tumor microenvironment and enhanced ICI efficacy.

### Butyrate enhances anti-tumor response by influencing the composition of the intestinal microbiome

**Figure 2.**
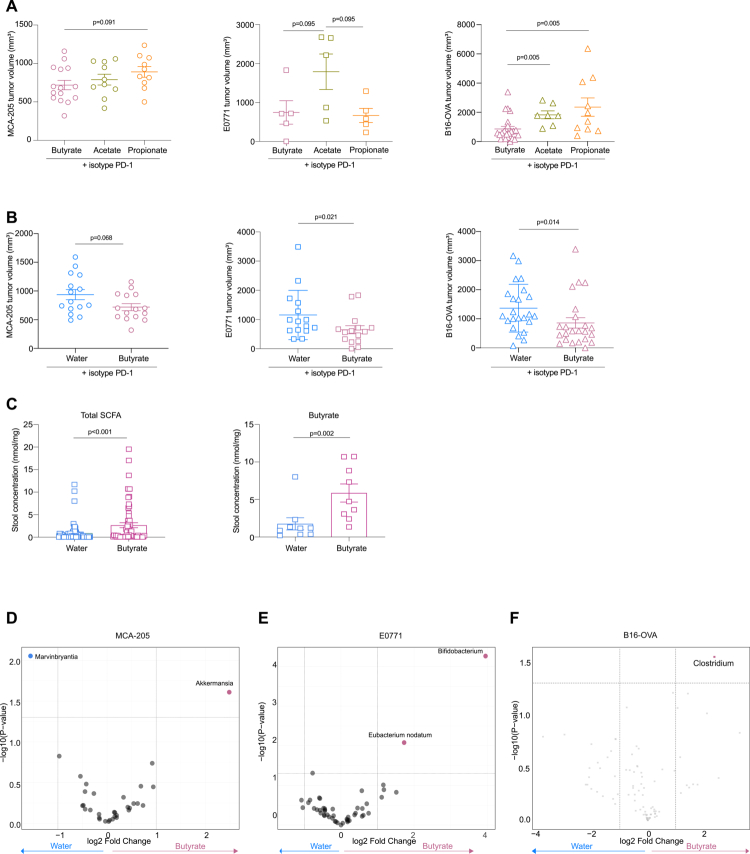
Butyrate influences the intestinal microbiome to enhance the anti-tumor response. (A) Tumor volume at sacrifice of subcutaneous MCA-205 (left), E0771 (middle), B16-OVA (right) implanted in mice supplemented with butyrate (respectively, *n* = 15; *n* = 5; *n* = 10), acetate (respectively, *n* = 10; *n* = 5; *n* = 7), or propionate (respectively, *n* = 10; *n* = 5; *n* = 10) in addition to an isotype control treatment. (B) Tumor volume at sacrifice of subcutaneous MCA-205 (left), E0771 (middle), B16-OVA (right) implanted in drinking water (respectively, *n* = 15; *n* = 15; *n* = 23) or butyrate (respectively, *n* = 15; *n* = 15; *n* = 23) mice in addition to an isotype control treatment. Results are shown from combined data from independent experiments, *n* refers to the number of mice analyzed per group. (C) Fecal concentration quantification of total SCFA representing the total number of individual metabolite measurements contributing to the calculated sum of SCFA analyzed (*n* = 63/groups, sum of acetate, butyrate, propionate, isobutyrate, isovalerate, succinate, lactate, and valerate), or butyrate from E0771 tumor-bearing mice after receiving either water or butyrate (*n* = 9/mice per group). Results are shown as mean ± SEM. Statistical significance was assessed using the Mann–Whitney U test. (D) Volcano plots illustrating the differential abundance of gut microbiome genera in response to butyrate treatment compared to water in MCA-205 bearing mice (water *n* = 20; butyrate *n* = 19), E0771 (water *n* = 18; butyrate *n* = 17) (E) and B16-OVA (water *n* = 10; butyrate *n* = 10). Results are shown from combined data from two independent experiments, *n* refers to the number of mice analyzed per group (F). The x-axis represents the log2 fold change in abundance of bacterial genera in fecal samples, with positive values indicating an increase with butyrate treatment. The y-axis represents the −log10 of the *p*-value, with higher values indicating greater statistical significance. Colored points denote genera with statistically significant changes (adjusted *p* < 0.05). Dashed lines indicate thresholds for statistical significance and fold change.

Subsequently, we sought to assess whether the anti-tumor effect of inulin could be replicated using microbiome-derived metabolites by administering the three most physiologically abundant SCFA. Butyrate, propionate, or sodium acetate were supplemented in the drinking water of mice at a concentration of 100 mM for 14 d before tumor inoculation with three different tumor models. SCFA supplementation was well tolerated with no observed side effects or weight loss, suggesting that palatability was not affected at this concentration (Supplementary Figure 2A). In the MCA-205, E0771, and B16-OVA tumor models, only butyrate administration was consistently associated with a significant reduction in tumor volume. Acetate showed no detectable anti-tumor effect, whereas propionate had mixed outcomes, with a decrease observed only in E0771 tumors (Figure 2A). We then confirmed the anti-tumor effect of butyrate by comparing its effect to water control mice in the three same tumor models and observed a significant reduction of tumor volume in the butyrate-supplemented mice (Figure 2B).

To assess the impact of butyrate supplementation on intestinal butyrate exposure and gut microbiome composition, we quantified fecal SCFA levels in butyrate-supplemented and water control mice. Oral butyrate supplementation significantly increased total SCFA and fecal butyrate concentrations compared to water control, confirming effective intestinal exposure (Figure 2C). Cecal butyrate concentrations also trended higher in the butyrate group, although this difference did not reach statistical significance (Supplementary Figure B). Moreover, oral supplementation of butyrate significantly increased the fecal concentration of other SCFA (Supplementary Figure 2C). We next performed 16S rRNA sequencing of fecal samples from the three tumor models, which showed that butyrate had no significant impact on alpha-diversity in the three tumor models (Supplementary Figure 2D). In the MCA-205 tumor model, we observed a significant difference in beta diversity and, at the taxa level, an enrichment of *A. muciniphila* (Figure 2D; Supplementary Figure 2E). In E0771, there was no significant difference in beta diversity but an upregulation of *Bifidobacterium* and *Eubacterium nodateum* compared to the control group (Figure 2E; Supplementary Figure 2E). Finally, in the B16-OVA model, we observed no differences regarding the beta-diversity, but an increase of *Clostridium* in the butyrate-supplemented mice (Figure 2F; Supplementary Figure 2E).

### The additive effect of butyrate and ⍺PD-1 implicates gut-homing markers

**Figure 3.**
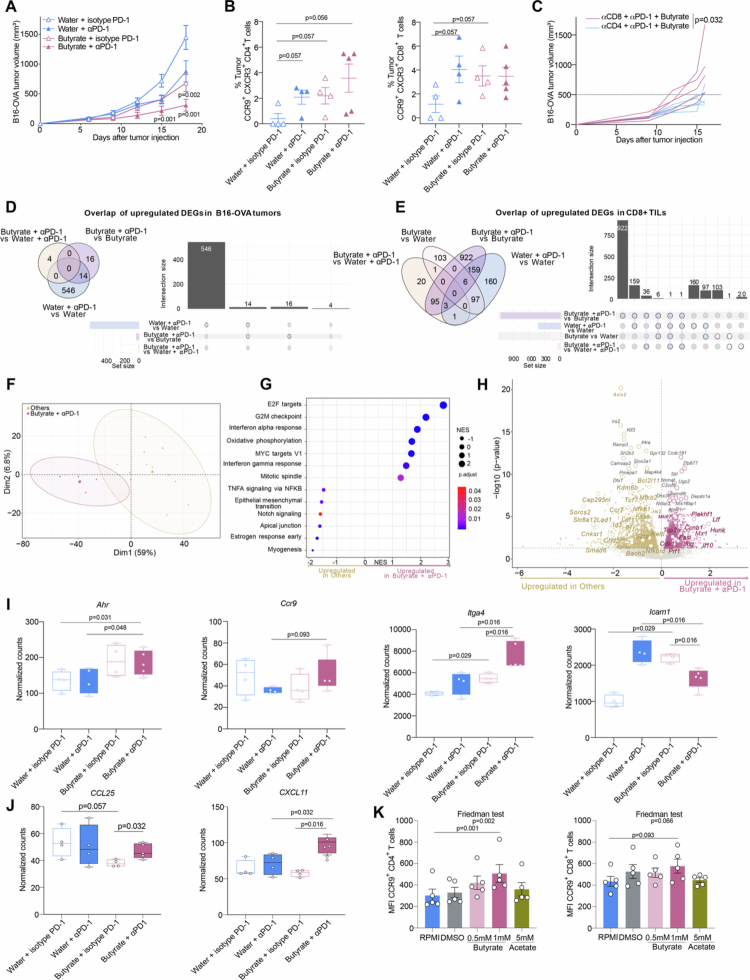
Butyrate influences metabolic and trafficking gene expression and promotes CD8-dependent anti-tumor response. (A) Tumor growth kinetics of subcutaneous B16-OVA tumor in mice receiving water (control) or butyrate supplementation, in the presence of an isotype control or **α**PD-1 treatment (*n* = 20 mice per group). Results are shown from combined data from four independent experiments. A mixed-effect analysis was conducted with time as a repeated measure. Treatment, including diet and iso/PD-1 treatment, was modeled as fixed effects, along with interaction terms to evaluate if growth curves separated over time. This analysis showed a significant effect of time (*p* < 0.001) and significant time × treatment (*p* < 0.001), indicating that tumor growth kinetics differed across diets and according to treatment over time. The main effect of treatment alone was not significant in this model (Treatment: *p* = 0.34). Selected cross-sectional comparisons (Wilcoxon tests), between water (isotype or **α**PD-1) and butyrate (isotype or **α**PD-1) at day 15 and day 18 are reported in the figure. (B) Flow cytometry analysis of intratumoral CCR9^+^ CXCR3^+^ CD4^+^ and CD8^+^ T cells frequency from B16-OVA tumor-bearing mice treated with either water or butyrate in the presence of an isotype or **α**PD-1 treatment (*n* = 4-5 mice per group). (C) Tumor growth kinetics of subcutaneous B16-OVA tumors in mice treated with anti–PD-1 in combination with butyrate and either anti-CD4 or anti-CD8 depleting antibodies (*n* = 5 mice/groups). Venn diagram and UpSet plot illustrating the overlap of upregulated differentially expressed genes (DEG) from bulk B16-OVA tumors (D) or CD8 + TILs (E) among indicated treatment comparisons (padj < 0.05 and FC > 1.25). Numbers represent unique or shared DEGs. (F) Principal component analysis (PCA) of CD8^+^ T cells transcriptomes from mice treated with anti–PD-1 or the pooled other groups. Each point represents an individual sample, and ellipses indicate 95% confidence intervals for each treatment group. (G) Gene Set Enrichment Analysis (GSEA) of CD8⁺ tumor-infiltrating lymphocytes from B16-OVA tumor-bearing, showing pathways enriched in mice treated with butyrate + anti–PD-1 vs. other (water + isotype, water + **α**PD-1, and butyrate + isotype). Dot plots represent normalized enrichment scores (NES) for selected pathways; dot size indicates NES magnitude, and the color scale represents adjusted *p*-values. (H) Volcano plot of differentially expressed genes in CD8⁺ tumor-infiltrating lymphocytes from B16-OVA tumor-bearing mice treated with butyrate + **α**PD-1 compared to the pool of control groups (water + isotype, water + **α**PD-1, and butyrate + isotype). Each dot represents one gene. The x-axis displays the log₂ fold change, and the y-axis displays the −log₁₀ *p*-value. Genes significantly upregulated in the Butyrate + **α**PD-1 group (log₂FC > 0, *p* < 0.05) are shown in pink, and genes significantly upregulated in the control pool (log₂FC < 0, *p* < 0.05) are shown in gold. Non-significant genes are shown in gray. (I) Normalized RNA-seq counts of *Ahr*, *Ccr9*, *Itga4*, and *Icam1* in CD8^+^ TILs from mice supplemented with water or butyrate and treated with either isotype control or anti–PD-1 antibody. (J) Normalized RNA-seq counts of *CCL25* and *CXCL11* in bulk B16-OVA tumors from mice supplemented with water or butyrate and treated with either isotype control or anti–PD-1 antibody. (K) Flow cytometry analysis of CCR9^+^ mean intensity fluorescence (MFI) on CD4⁺ and CD8⁺ T cells stimulated in vitro in the presence of RPMI, DMSO, butyrate (0.5 or 1 mM), or Acetate (10 mM). Data represent pooled results from 5 donors. Results are shown as mean ± SEM. Statistical significance was assessed using Friedman's test, followed by Dunn's multiple comparison test.

To characterize the effect of butyrate on ⍺PD-1 response, we focused on the B16-OVA model, which displayed the most pronounced tumor decrease among the tested models treated with butyrate (Figure 2A, B). While butyrate alone significantly reduced tumor growth, its combination with ⍺PD-1 produced an additive anti-tumor effect compared with ⍺PD-1 treatment alone (Figure 3A).

To further dissect the immunological impact of butyrate in the observed anti-tumor response, we employed three different complementary approaches. First, flow-cytometry analysis revealed an increase in intratumoral CCR9⁺CXCR3⁺ cells in the butyrate + ⍺PD-1 group within the CD4⁺ T cells population, and within the CD8⁺ T cells population in the butyrate mice group (Figure 3B). Moreover, an upregulation of CCR9^+^CXCR3^+^ CD4^+^ and CD8^+^ T cell populations in the spleens of mice receiving butyrate in addition to ⍺PD-1 treatment was observed (Supplementary Figure 3A). These results mirrored the immune shifts in CCR9^+^ CD4^+^ and CCR9^+^ CD8^+^ T cells observed in the inulin experiments.

Second, depletion experiments using anti-CD8 or anti-CD4 antibodies were performed in B16-OVA tumor-bearing mice treated with ⍺PD-1 that received butyrate supplementation. Depletion of CD8⁺ T cells abrogated the anti-tumor effect of butyrate, whereas CD4⁺ T cell depletion did not impair tumor control (Figure 3C).

Third, we performed RNA sequencing transcriptome profiling on bulk B16-OVA tumors and in tumor-infiltrating CD8^+^ T cells (TILs) in mice treated with or without butyrate supplementation, in combination with either isotype or ⍺PD-1 therapy. To provide a comprehensive view of transcriptional differences across treatment comparisons, we examined both upregulated and downregulated differentially expressed genes (DEGs) in bulk tumors and in CD8^+^ TILs using combined Venn diagrams and UpSet plots. In bulk tumors, the dominant transcriptional effect was driven by **α**PD-1 alone, with the largest gene sets uniquely associated with the water + **α**PD-1 vs. water comparison in both upregulated and downregulated DEGs, and minimal overlap across the other comparisons (Figure 3D; Supplementary Figure 3B). In contrast, in CD8⁺ TILs, the largest unique gene sets were consistently identified in the butyrate + **α**PD-1 vs. butyrate comparison, indicating that butyrate substantially amplified the transcriptional response to **α**PD-1 specifically within CD8⁺ TILs rather than in the bulk tumor (Figure 3E; Supplementary Figure 3C).

To estimate tumor immune infiltration, we used the Immune Cell Abundance Identifier (ImmucellAI) tool on the bulk tumor transcriptomic data.^32^ This analysis revealed an increase of CD8^+^ T cells and, more specifically, cytotoxic and central memory CD8^+^ T cells in the butyrate + ⍺PD-1 tumor group (Supplementary Figure 3D). In contrast, there was no increase in CD4^+^ T cells or CD4^+^ regulatory T cells in the butyrate ⍺PD-1 group (Supplementary Figure 3E), further demonstrating the importance of CD8⁺ T cell activity in butyrate anti-tumor-mediated response.

To further characterize the transcriptional reprogramming induced by butyrate in the context of ICI, we performed unsupervised analyses comparing the butyrate + **α**PD-1 group against the pool of the other conditions in CD8⁺ TILs. PCA revealed a clear separation of butyrate + **α**PD-1 samples from all other groups along the first principal component (Figure 3F). Gene Set Enrichment Analysis (GSEA) identified 13 significantly enriched pathways. Pathways enriched in the butyrate + **α**PD-1 group included cell cycle progression (E2F targets, G2M checkpoint), metabolically active (Oxidative phosphorylation), and interferon-responsive (Interferon-*γ* response) CD8⁺ T cell phenotype. Conversely, pathways enriched in the “others” group included proinflammatory (TNF-***α*** signaling via NFkB), tissue remodeling (Epithelial-mesenchymal transition), and developmental (Notch signaling) function (Figure 3G). To provide a gene-level resolution of these transcriptional differences, we generated a volcano plot displaying all genes tested in the butyrate + **α**PD-1 compared to the pool of other conditions (Figure 3H). The most statistically significant genes on each side are annotated, with biologically relevant genes highlighted in bold. In the butyrate + **α**PD-1 group, upregulated genes included markers of T cell proliferation (*Mki67*, *Ccnb1*, *Cdk1*), and effector function (*Ifng*, *Prf1*, *Fasl*). In the group “others”, upregulated genes included markers of stem-like and central memory T cell program (*Tcf7*, *Ccr7*, *Id3*) and NF-κB signaling (*Nfkb1*, *Nfkb2*, *Relb*).

Given the known ability of butyrate to influence cellular metabolism, we next performed a targeted analysis of genes involved in metabolic regulation and T cell trafficking on B16-OVA tumor-infiltrating CD8⁺ T cells. Unsupervised hierarchical clustering of samples confirmed that the experimental condition drove gene expression patterns, with butyrate + **α**PD-1 samples clustering distinctly from the “others” groups (Supplementary Figure 3F). Among metabolic regulators, we observed a trend in butyrate + ⍺PD-1 toward increased expression of the aryl hydrocarbon receptor (*Ahr*), consistent with butyrate's reported ability to modulate this receptor signaling^37^ ^,^ ^38^ (Figure 3I; Supplementary Figure 3F). While the glycolytic enzyme *Hk2* was significantly downregulated in CD8^+^ T cells from butyrate-supplemented mice (Supplementary Figure 3F). We then examined genes associated with trafficking and adhesion. Both water + ⍺PD-1 and butyrate + ⍺PD-1 groups displayed reduced expression of *Ccr7* and *S1pr1* (Supplementary Figure 3F). In contrast, butyrate + ⍺PD-1 supplementation led to a numerical increase in *Ccr9* and a significant increase in *Itga4*. *Icam1* expression was significantly increased in the butyrate group compared with water, but decreased in the butyrate + ⍺PD-1 condition compared with water + ⍺PD-1 (Figure 3I).

Moreover, to evaluate whether butyrate-driven transcriptional changes in the tumor were accompanied by corresponding ligand expression in the tumor microenvironment, we interrogated our bulk B16-OVA tumor RNA-seq dataset for expression of relevant chemokine ligands. *CCL25*, the cognate ligand for *CCR9*, was significantly reduced in butyrate compared to water, but was increased in butyrate + **α**PD-1 tumors compared with butyrate alone (Figure 3J). *CXCL11*, a ligand of *CXCR3*, was increased in butyrate + **α**PD-1 compared with butyrate alone and water + **α**PD-1 (Figure 3J). In contrast, the other *CXCR3* ligands, *CXCL9* and *CXCL10*, were increased in the presence of **α**PD-1 irrespective of butyrate supplementation (Supplementary Figure 3G).

Given the observed impact of butyrate on T cell trafficking markers in vivo, we then investigated its effect on modulating homing receptor expression in human T cells by culturing CD4⁺ and CD8⁺ T cells isolated from peripheral blood mononuclear cell (PBMCs) of healthy donors in the presence of butyrate or acetate. While butyrate did not significantly change the frequency of CCR9⁺ T cells, we observed a significant increase in CCR9 mean fluorescence intensity (MFI) in CD4⁺ and CD8^+^ T cells stimulated with 1 mM butyrate (Figure 3K, Supplementary Figure 3H). In the case of integrin **α**4β7, butyrate addition led to a concentration-dependent trend toward reduced frequency (Supplementary Figure 3I-J). Because *AhR* expression was upregulated in T cells from butyrate-treated mice, and previous studies have shown that AhR can regulate markers involved in lymphocyte trafficking, such as integrin β7,^39^ we hypothesized that the effects of butyrate on homing receptor expression might be mediated through AhR signaling. Therefore, we co-cultured CD4^+^ and CD8^+^ T cells together with the AhR agonist 6-formylindolo[3,2-b]carbazole (FICZ) or the antagonist CH-223191 (CH22) to determine whether these effects were AhR-dependent. CCR9 expression was not significantly influenced by AhR modulation in either CD4⁺ or CD8⁺ T cells (Supplementary Figure 3K). In contrast, co-culture with CH22 significantly reduced integrin **α**4β7 expression, supporting AhR involvement in its regulation (Supplementary Figure 3L).

Together, these results demonstrate that butyrate enhances the anti-tumor efficacy of **α**PD-1 through reprogramming of CD8⁺ T cells to promote proliferative and metabolic pathways as well as trafficking markers.

### Dietary fiber did not correlate with the outcome of NSCLC patients treated with ICI

Considering the recent link between diet and outcome of patients with melanoma treated with ICI, we prospectively collected dietary surveys using a modified NIH food frequency questionnaire (FFQ) in 117 patients with advanced NSCLC treated with single-agent ⍺PD-1 or in combination with chemotherapy. The median age of the cohort was 72 y, and it was composed of 53% females and 47% males, with 38% receiving ⍺PD-1 alone. The median follow-up was 14 months. Complete cohort characteristics are summarized in Supplementary Table 2.

**Figure 4.**
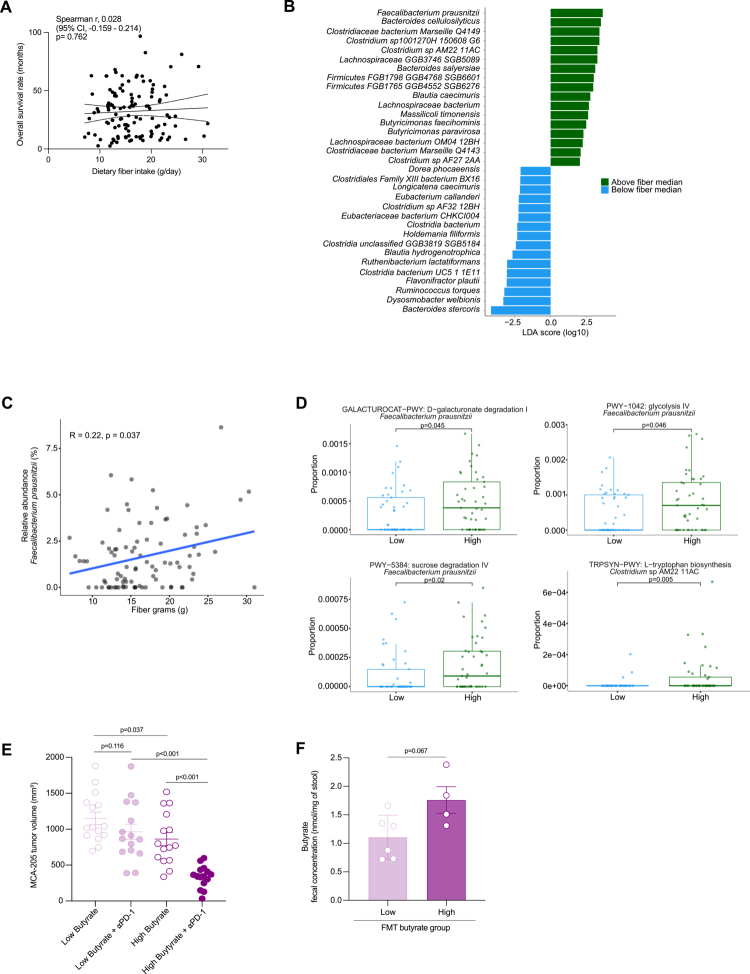
Fiber intake does not correlate with clinical outcome of NSCLC, but is associated with enrichment of butyrate-producing bacteria. (A) Scatter plot showing the correlation between total dietary fiber intake (g/d) and overall survival (months) in NSCLC patients (*n* = 117). (B) Linear discriminant analysis (LDA) effect size (LEfSe) plot assessed by shotgun metagenomics showing the differentially abundant bacterial taxa in the gut microbiota of individuals above or below the median of dietary fiber intake (g/d). The x-axis represents the LDA score (log 10), indicating the magnitude of the difference in relative abundance. (C) Spearman correlation between the relative abundance of *F. prausnitzii* (percentage) and fiber intake (grams) in NSCLC patients. (D) Relative abundance of MetaCyc pathways attributed to *F. prausnitzii* in fecal metagenomes from individuals with above or below median fiber intake. (E) Tumor volume at sacrifice from subcutaneous MCA-205 tumor mice experiment after fecal microbiota transplantation (FMT) from low vs. high butyrate stool patients' concentration, in the presence of an isotype control or **α**PD-1 treatment (*n* = 15 mice/groups). (F) Mice fecal butyrate concentration from MCA-205 tumor experiment after fecal microbiota transplantation (FMT) from low vs. high butyrate stool patients' concentration. Data are shown as mean ± SEM. A Mann–Whitney test was used to compare differences between groups.

There was no correlation between fiber intake and improvement in overall survival (OS) or progression-free survival (PFS) (Figure 4A; Supplementary Figure 4A). To account for potential cofounders, we performed a Cox proportional hazard model including smoking status, ECOG, PD-L1 category, stage, sex, and treatment regimen (anti–PD-1 alone vs. combination). In these adjusted models, only PDL-1 expression was significantly associated with outcome (OS: HR 2.34, 95% CI 1.14–4.78; *p* = 0.02) in line with prior studies.^40^ ^,^ ^41^ However, dietary fiber intake was not associated with OS (HR 1.01, 95% CI 0.93–1.09; *p* = 0.81) or PFS (HR 1.01, 95% CI 0.95–1.08 *p* = 0.73) (Supplementary Figure 4B). Of note, the median dietary fiber intake was 15.6 g/d, ranging from 7.1 to 31 g/d, resulting in 59%–76% patients below previously published thresholds (17 g/d; 20 g/d)^16^ ^,^ ^42^ (Supplementary Figure 4C).

We then compared the gut microbiome of patients stratified according to their median daily fiber intake. Among the 117 patients, shotgun metagenomic sequencing was available and performed in 90 patients. The analysis revealed a numerical increase in the alpha but no differences in beta-diversity in the above fiber median group compared to the below fiber median group. (Supplementary Figure 4D-E). At the taxa level, a higher abundance of *F. prausnitzii*, *Bacteroides cellulosilyticus*, *Clostridium* sp., *Lachnospiracea,* and *Butyricimonas* sp. was observed in the above fiber median group compared to the below fiber median group (Figure 4B). Spearman's correlation analysis confirmed a significant positive association between fiber intake and the relative abundance of *F. prausnitzii* (Figure 4C). Next, we assessed the pathway related to our metagenomics data. Functional profiling using MetaCyc metabolic pathway analysis showed a significant enrichment of several microbial metabolic pathways in the above fiber median group including sucrose degradation and tryptophan biosynthesis, with contributions primarily from *F. prausnitzii* and *Clostridium* sp. (Figure 4D).

Lastly, we performed targeted quantification of SCFA in the patients' fecal samples and did not observe a correlation between individual SCFA and the above vs. below fiber median groups (Supplementary Figure 4F). Despite the absence of correlation, we asked whether donor stools stratified by fecal butyrate concentration could transfer a similar metabolic function to recipient mice after FMT. We selected 6 patients with either low (*n* = 3) or high (*n* = 3) fecal butyrate concentrations; their clinical characteristics are summarized in Supplementary Table 3. Following FMT into antibiotic-treated MCA-205 tumor-bearing mice housed under SPF conditions, recipients of “high-butyrate” donor exhibited significantly reduced tumor growth, an effect that was further enhanced in combination with **α**PD-1 treatment (Figure 4E). Moreover, transfer of feces from donor high-butyrate group was reflected in the metabolic output of recipient mice post-FMT, with higher levels of butyrate in the mice feces at sacrifice compared to mice transferred with low-butyrate donors (Figure 4F). These data indicate that fecal material transfer from donors with high butyrate concentration can reproduce a butyrate-associated function in recipient mice and is linked to improved tumor control.

Taken together, these findings indicate that although fiber intake was not correlated with clinical outcomes in our cohort, likely reflecting the generally low fiber intake among cancer patients, it remained associated with specific beneficial microbiome characteristics, particularly enrichment in *F. prausnitzii* and metabolic pathways.

## Discussion

This study investigates the relationship between fermentable fiber intake, microbial metabolites, and anti-tumor immunity in the context of ICI therapy. While the ability of inulin to modulate gut microbiome composition and SCFA production has been previously described, our study adds several observations. First, our data indicate that inulin supplementation reshapes the gut microbiome, whereas butyrate supplementation appears to exert its anti-tumor effects without inducing major changes in microbiome composition, suggesting that these interventions may act at different levels of the gut microbiome–immune axis. Second, butyrate supplementation improved ICI response in a CD8⁺ T cell-dependent manner and was associated with transcriptional remodeling of tumor-infiltrating CD8⁺ T cells. Third, both inulin and butyrate increased the frequency of T cell populations expressing markers associated with intestinal homing and inflammatory trafficking, including CCR9 and CXCR3, suggesting a potential role for microbial metabolites in shaping T cell trafficking. Finally, we assessed dietary fiber intake in patients with NSCLC treated with ICI, a clinical population in which this question remains poorly characterized. Together, these findings support a model in which fermentable fibers may act upstream by remodeling the gut microbiome and SCFA production, whereas butyrate may act downstream as a microbial metabolite capable of modulating CD8⁺ T cell function and trafficking, although the underlying mechanisms require further validation.

Diet is a major determinant of the composition and metabolic function of the gut microbiome. Nutritional intervention studies have demonstrated that the gut ecosystem can undergo significant changes within 24 hours following substantial dietary modifications.^43^ ^,^ ^44^ One important message from our study, together with recent preclinical studies, is that inulin supplementation can reshape the gut microbiome and improve anti-tumor responses in mice, although the bacterial taxa enriched after inulin differ between studies. We observed an enrichment in *Akkermansia*, *Lachnospiraceae*, and *Alistipes* bacteria. Li et al., recently reported an increase in the butyrate-producing *Clostridium* XIVa cluster, and Boucher et al. reported a predominance of *Bifidobacterium.* ^14^ ^,^ ^15^ These differences may reflect mouse housing conditions, animal providers or the structural complexity of the fiber source, as proposed by Hamaker and Tuncil's hierarchical model.^45^ Inulin, for example, is a simple linear structure composed of fructose units and a terminal glucose residue.^46^ This structural simplicity makes it accessible to a wide range of gut bacteria, reducing competition between taxa and leading to variable responses depending on the initial microbial composition.^45^ Despite these differences in intestinal microbial composition, there appears to be a convergent agreement on the anti-tumor potential of fermentable fibers in preclinical settings. Indeed, their potential has already been demonstrated in mouse models carrying melanocytic (YUMM) or colonic (MC38) tumors that were fed a diet rich in inulin and mucin, resulting in a significant reduction in tumor size, dependent on CD8⁺ T lymphocytes.^15^ In other models, it has been reported that an inulin-enriched diet increased anti-tumor responses in three tumor models, including B16-OVA and MCA-205, through the activation of CD4⁺ Th1-polarized T lymphocytes and CD8⁺ **α**β T lymphocytes.^14^

In humans, observational studies have shown that a plant-based diet is associated with greater bacterial diversity, as well as increased abundance of *Bifidobacterium*, *Lactobacillus*, and *Ruminococcus,* taxa repeatedly linked with positive response to ICI.^47-49^ Spencer et al. previously showed that melanoma patients treated with **α**PD-1 with *sufficient* fiber intake (>20 g/d) had improved progression-free survival compared to those with insufficient intake, with a 30% reduction in the risk of progression or death for each 5 g increase in fiber.^16^ Moreover, a recent meta-analysis of 310 patients from four independent studies confirmed this observation, linking high fiber intake to favorable clinical outcomes in cancer patients receiving ICI therapy.^50^

In our NSCLC cohort, fiber intake did not correlate with clinical outcomes, which may reflect the low overall fiber consumption among cancer patients. This low consumption may have limited our ability to detect associations between fiber intake and response to ICI. Consistent with our observations, no significant differences in overall microbiome composition were detected between these patient groups, apart from an enrichment of *F. prausnitzii* in the high-fiber group. This species is one of the main butyrate-producing commensals in the human gut microbiota and is widely recognized as a marker of gut health and anti-inflammatory capacity.^51^ These findings suggest that consumption of fermentable fiber, even at modest levels, may still promote the growth of beneficial SCFA-producing taxa. To our knowledge, this is the first study to assess dietary intake in patients with NSCLC treated with ICI. Previous studies, particularly in melanoma, have linked higher dietary fiber intake with improved ICI outcomes. Our work extends this question to NSCLC, a population in which dietary intake and microbiome-metabolite interactions remain less characterized.

Based on the results from our study, our group initiated a randomized clinical trial in patients with NSCLC treated with ICI (NCT05805319). In this study, patients receive either standard ICI therapy alone or in combination with a nutrition intervention supervised by a dietitian aimed at increasing total fiber intake. The goal of this intervention is to gradually increase daily fiber consumption to more than 25 g/d. Targeted dietary intervention, such as proposed in this clinical trial, could represent a realistic strategy for supporting anti-tumor responses.

The results of our study revealed the potential of butyrate as an important immunomodulatory metabolite capable of modulating ICI responses in preclinical models. Among the three main SCFA tested, butyrate showed the most consistent association with reduced tumor growth in several models. In line with our findings, butyrate has previously been recognized as promoting the activation of effector and memory CD8⁺ T cells by reprogramming their metabolism and epigenome, notably via histone deacetylase (HDAC) inhibition and mTOR pathway activation.^21-23^ Although butyrate is known to induce regulatory T cell differentiation, we did not observe FoxP3⁺ CD4⁺ T cell expansion in our supplemented mice.^52^ ^,^ ^53^ Recent studies have shown that butyrate-dependent CD8⁺ action leads to a more effective response to cancer treatment. He et al., first demonstrated that butyrate supplementation alone can restore the effect of cisplatin therapy in mice treated with antibiotics and that this action requires the presence of CD8^+^ T cells, as depletion of these cells, but not CD4^+^ T cells, leads to a loss of the efficacy of butyrate supplementation, as observed in our results.^22^ Subsequently, two studies showed that this beneficial action of butyrate is also observed in the context of cancer immunotherapy, demonstrating that the addition of butyrate induced an increase in the expression of *PD1* and *CD28* through epigenetic modification, as well as the production of TNF-***α*** and IFN-*γ* by cytotoxic CD8^+^ T cells, resulting in a significant reduction in mouse tumors.^54^ ^,^ ^55^ This discordance in the induction of regulatory or effector profiles appears to be concentration-dependent. A study has shown that at low concentrations, butyrate promotes T regulatory cell differentiation, while at higher concentrations it induces Th1 and cytotoxic profiles.^56^ However, in our in vitro experiments, as in others, concentrations above 1 mM proved to be toxic, leading to cell death. This limit also illustrates the difficulty of translating the human luminal butyrate concentrations (10-20mM) into in vitro models.

Beyond the activation of effector markers, we observed in vivo an increase in the frequency of CCR9⁺CXCR3⁺ T cells in both tumors and spleens of butyrate-treated mice. The origin and programming of this population, however, remains an open question that our study was not designed to resolve, and which will require dedicated experiments to address. CCR9 expression is generally regarded as indicative of an intestinal imprint, while CXCR3 is known to be expressed on activated Th1 cells.^57^ ^,^ ^58^ Whether the CCR9⁺ CXCR3⁺ T cells phenotype observed here reflects cells that were gut-primed and subsequently co-opted CXCR3 expression upon encountering inflammatory signals in the tumor, or that they acquired both receptors through the local tumor microenvironment, cannot be determined from our data alone. Several elements from our study could be interpreted as supporting a gut-imprinting hypothesis. The enrichment of CCR9⁺CXCR3⁺ T cells in the spleen could suggest systemic reprogramming of the T cell pool, but splenic enrichment does not necessarily imply intestinal origin, as butyrate can potentially reach systemic circulation after oral administration and act directly on T cells in secondary lymphoid organs. Our in vitro data showing that butyrate increases CCR9 MFI on human T cells supports the possibility of a direct effect of butyrate on CCR9 expression, but in vitro stimulation of peripheral T cells does not recapitulate the intestinal priming microenvironment and cannot prove that this imprinting occurs in the gut in vivo.

Regarding the tumor microenvironment, interrogation of our bulk B16-OVA tumor RNA-seq dataset revealed that *CCL25* was significantly downregulated in butyrate + isotype control tumor groups. However, *CCL25* trended toward restoration in butyrate + **α**PD-1 tumors, while *CXCL11* was elevated by the combination. *CXCL9* and *CXCL10* were upregulated by **α**PD-1 independently of butyrate, consistent with published data on their role in **α**PD-1 response.^59^ These data raise the possibility that the combination of butyrate and **α**PD-1, rather than either treatment alone, may create a chemokine landscape permissive for CCR9⁺CXCR3⁺ T cell recruitment, but whether these cells arrive from the gut, the systemic circulation, or are locally imprinted remains to be determined.

One potential molecular mechanism through which butyrate could influence T cell trafficking is through the aryl hydrocarbon receptor (AhR). In CD8⁺ TILs from butyrate-treated mice, we observed an upregulation of Ahr expression, consistent with previously reported links between butyrate and AhR signaling. The AhR has initially been characterized as an environment-sensitive transcription factor, initially described for its strong affinity to the organic pollutant 2,3,7,8-tetrachlorodibenzo-*p*-dioxin (TCDD).^60^ It is now recognized that, in addition to environmental factors, dietary and microbial-derived molecules, particularly from tryptophan metabolism, can also activate AhR. Furthermore, recent articles have established a link between butyrate and AhR expression, reinforcing the potential link between the two in the expression of CCR9 and other homing markers.^37^ ^,^ ^38^ However, in vitro observations appear more mixed. In our experiments, stimulation of human CD4⁺ and CD8⁺ lymphocytes in the presence of butyrate did not alter the frequency of CCR9⁺ cells. Still, it did result in a significant increase in the MFI of this marker. In contrast, the expression of ***α**4β7* integrin showed a dose-dependent reduction, suggesting distinct effects of butyrate on these two axes of intestinal homing. Consistent with this, other studies conducted on human PBMC have reported a reduction in *CCR9* expression after butyrate stimulation.^35^ Moreover, when we evaluated in vitro the involvement of AhR in these effects, neither the agonist FICZ nor the antagonist CH-223191 altered *CCR9* expression, while pharmacological blockade of AhR reduced integrin ***α**4β7* expression, suggesting partial and selective regulation of these adhesion molecules by AhR. These observations are in keeping with those of Qiang et al., who reported that butyrate potentiates the action of RA on integrin **α**4β7 induction, but without a significant effect on CCR9.^61^ Also, the divergence between in vivo and in vitro results suggests that butyrate may act in a complex ecosystem, interacting with the microbiome, CD103⁺ dendritic cells, and various microbial metabolites that activate AhR. These elements may converge to create a microenvironment permissive for the induction of CCR9, integrin **α**4β7, and other markers of the same type. Conversely, in vitro, in the absence of these environmental signals, butyrate may exert its direct epigenetic or GPCRs activation effects, which may lead to a lack of induction or even a reduction in certain homing markers, as observed in our results and those of other teams. Formally establishing the gut-priming origin of these cells would require dedicated T-cell tracking experiments, adoptive transfer of gut-derived CCR9⁺ T cells, or paired TCR clonotype analysis between gut-associated lymphoid tissue and tumor compartments. These mechanistic questions represent a priority for future investigations, as understanding the origin of this population could have important implications for the development of microbiome- or dietary-based strategies to improve T cell recruitment to tumors.

In humans, clinical trials using butyrate remain limited and heterogeneous, but support its potential value as a metabolic and anti-inflammatory modulator. Recent studies, particularly in pediatric obesity and ulcerative colitis, have shown that butyrate supplementation can reduce systemic inflammation and improve certain metabolic parameters without major adverse effects.^62^ ^,^ ^63^ Other studies, such as the AusFAP trial using butyrate-enriched resistant starch for the prevention of colorectal cancer, support the feasibility of approaches aimed at increasing endogenous SCFA production.^64^ However, no clinical trials have yet evaluated butyrate supplementation in the context of ICI therapy.

In conclusion, our results support a model in which fermentable fibers may contribute to improved **α**PD-1 responses through modulation of the gut microbiome, butyrate production, and immune trafficking pathways. These findings provide new insights for the development of nutritional or microbiome-based interventions aimed at supporting cancer immunotherapy, while emphasizing the need for further studies to establish causality and further validate these mechanisms.

## Supplementary Material

Supplementary Material Final_figures_02Apr

Supplementary_captions.docx

## Data Availability

The sequencing datasets analyzed during the current study are available in the NCBI Sequence Read Archive (SRA) under BioProject accession number PRJNA1393370 (https://dataview.ncbi.nlm.nih.gov/object/PRJNA1393370?reviewer=u9hge9mveca288utriu43f63t2). No custom code or scripts were used in this study. All analyses were performed using standard publicly available software, as detailed in the Methods section.
