# Supplementary figures and images for: Dietary intervention through bacterial-derived butyrate elicits anti-tumor activity and increases anti-PD-1 response
Source: Gut Microbes. 2026 Jul 15;18(1):2699457. doi: 10.1080/19490976.2026.2699457 (PMC13374751; doi:10.1080/19490976.2026.2699457)

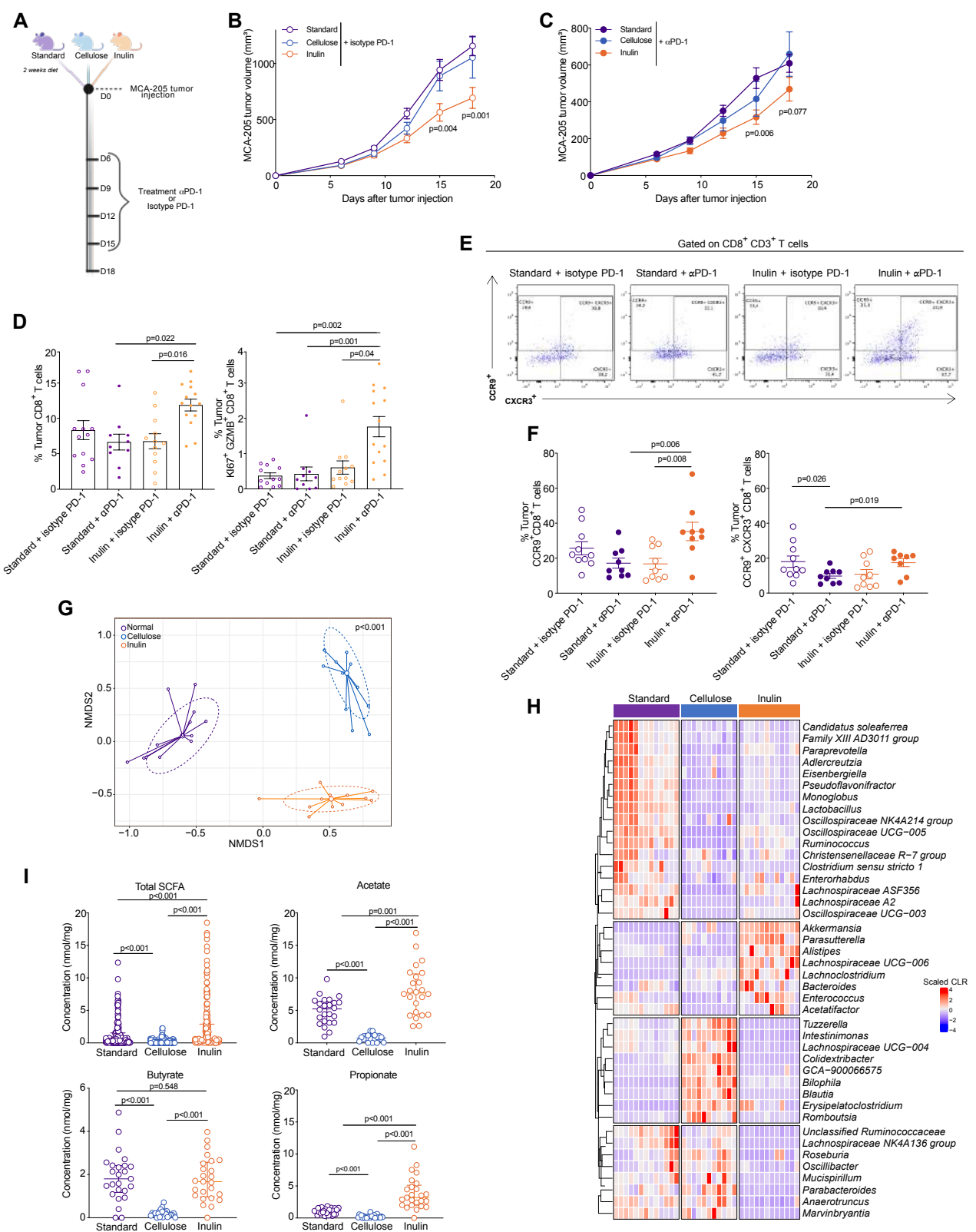

**FIGURE 1**

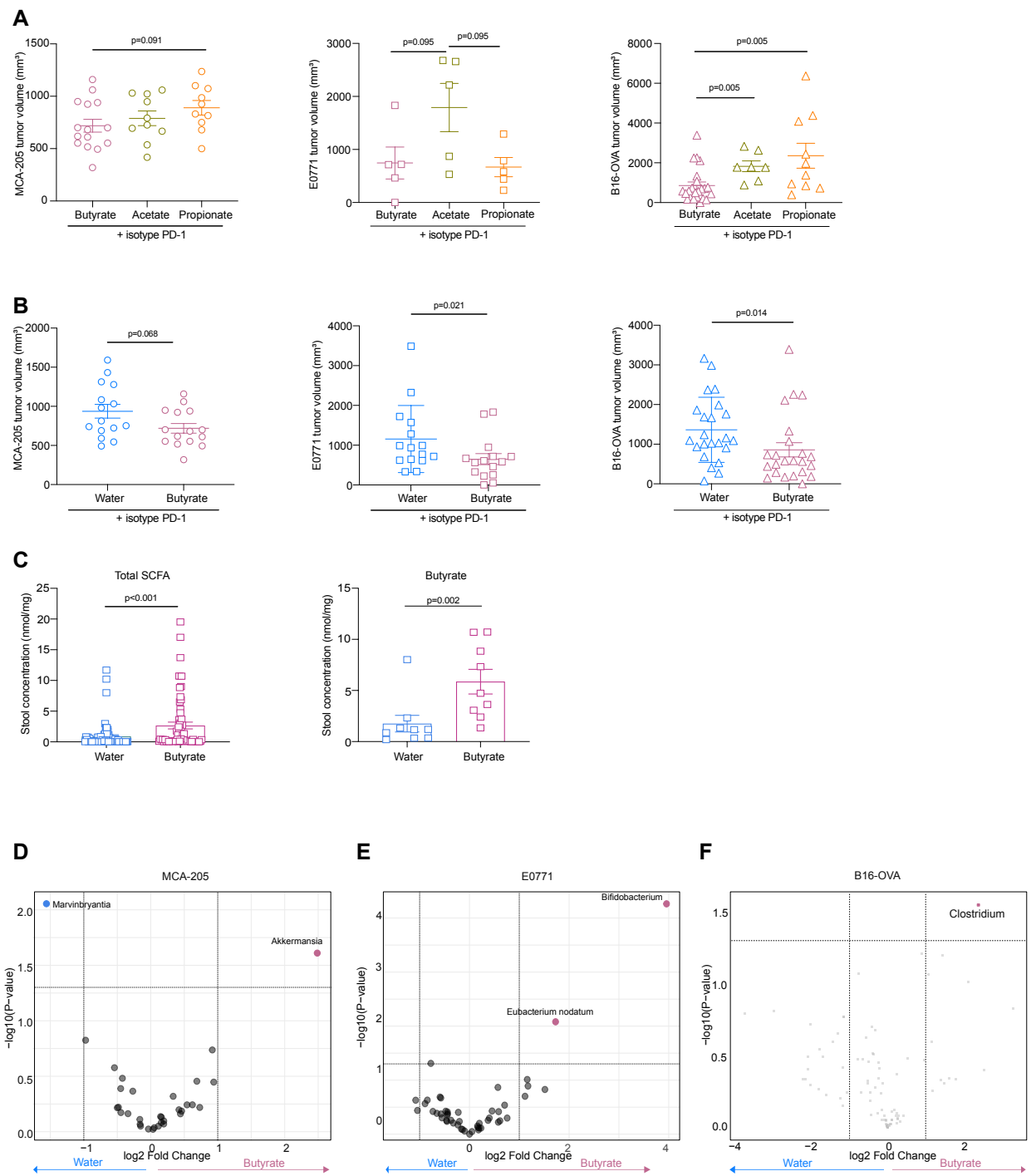

**FIGURE 2**

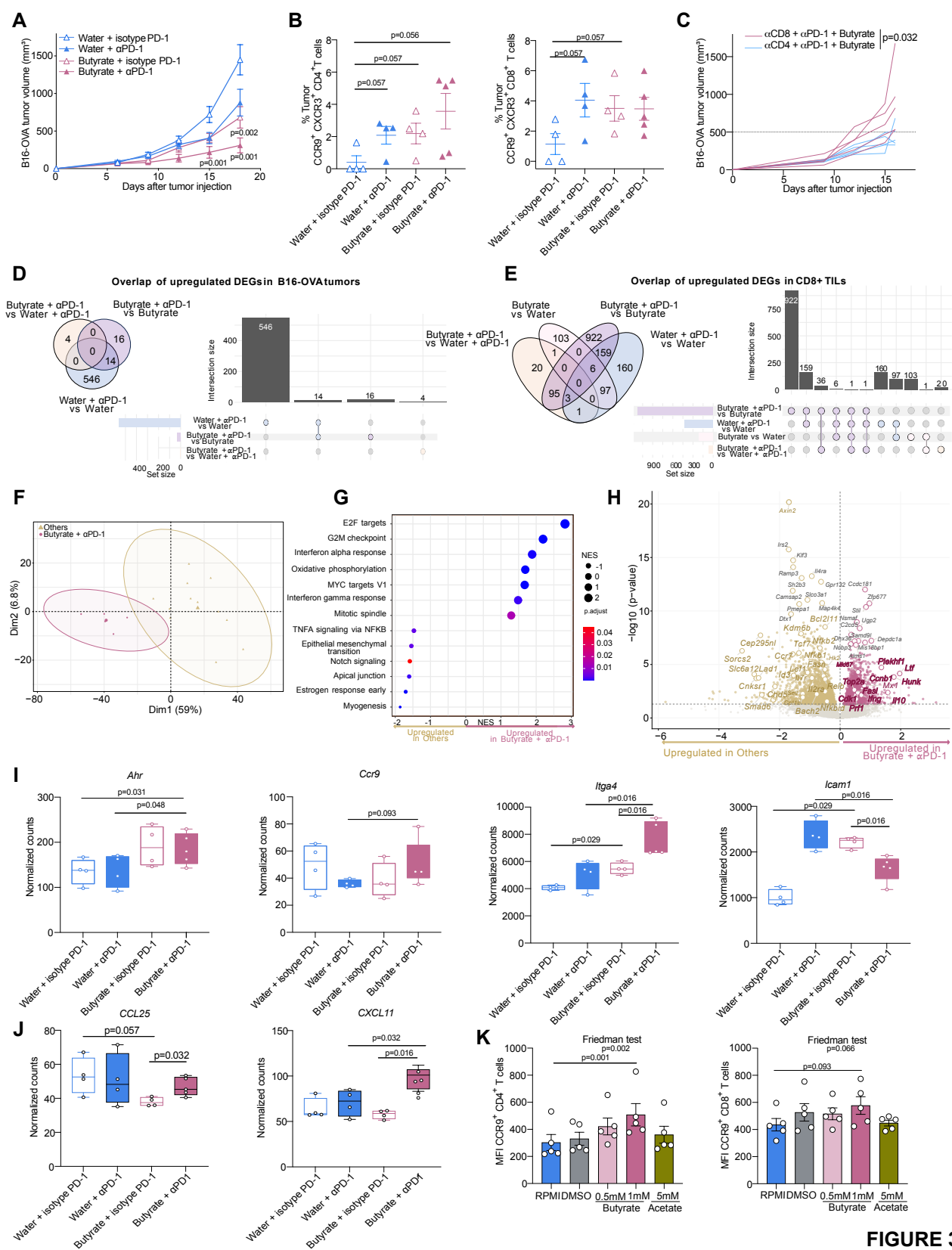

**FIGURE 3**

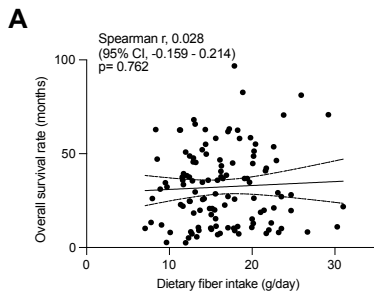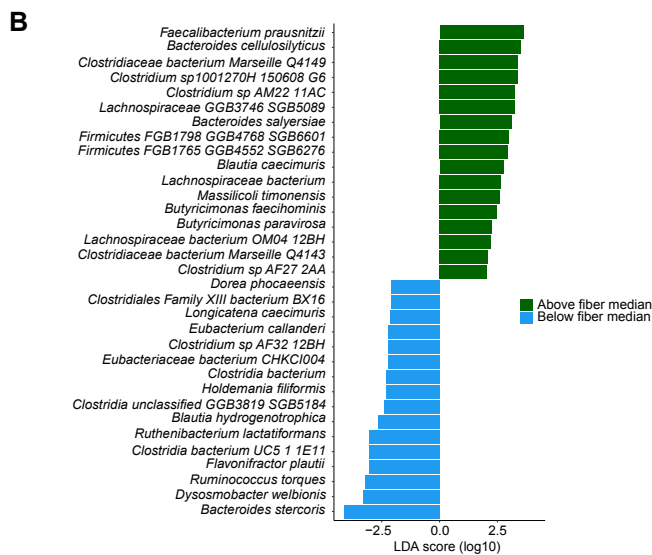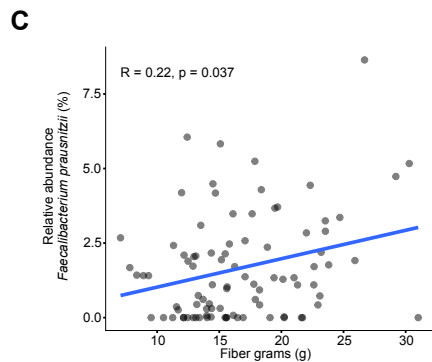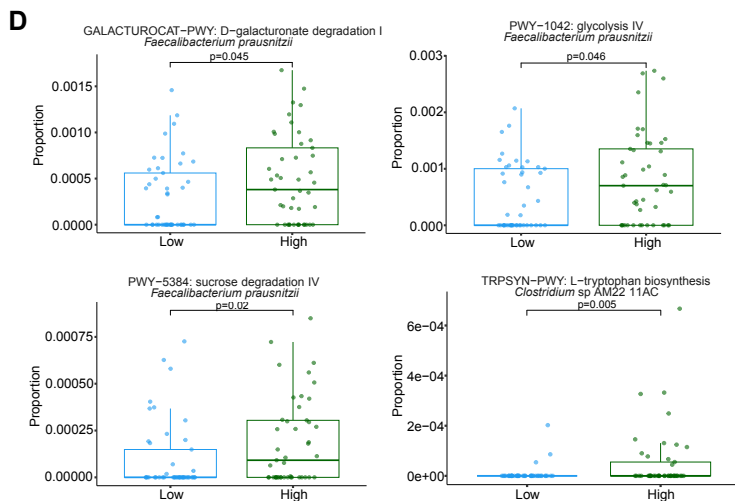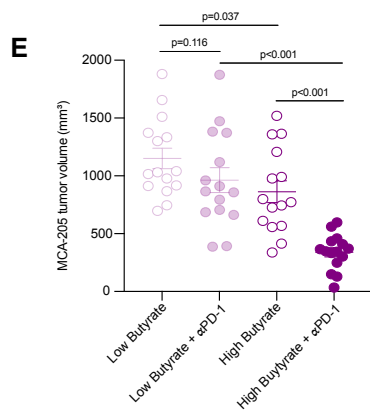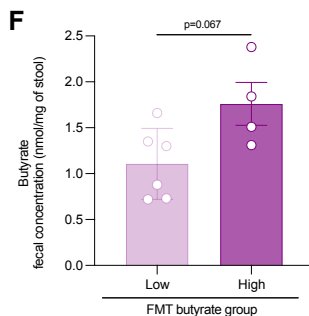

**FIGURE 4**

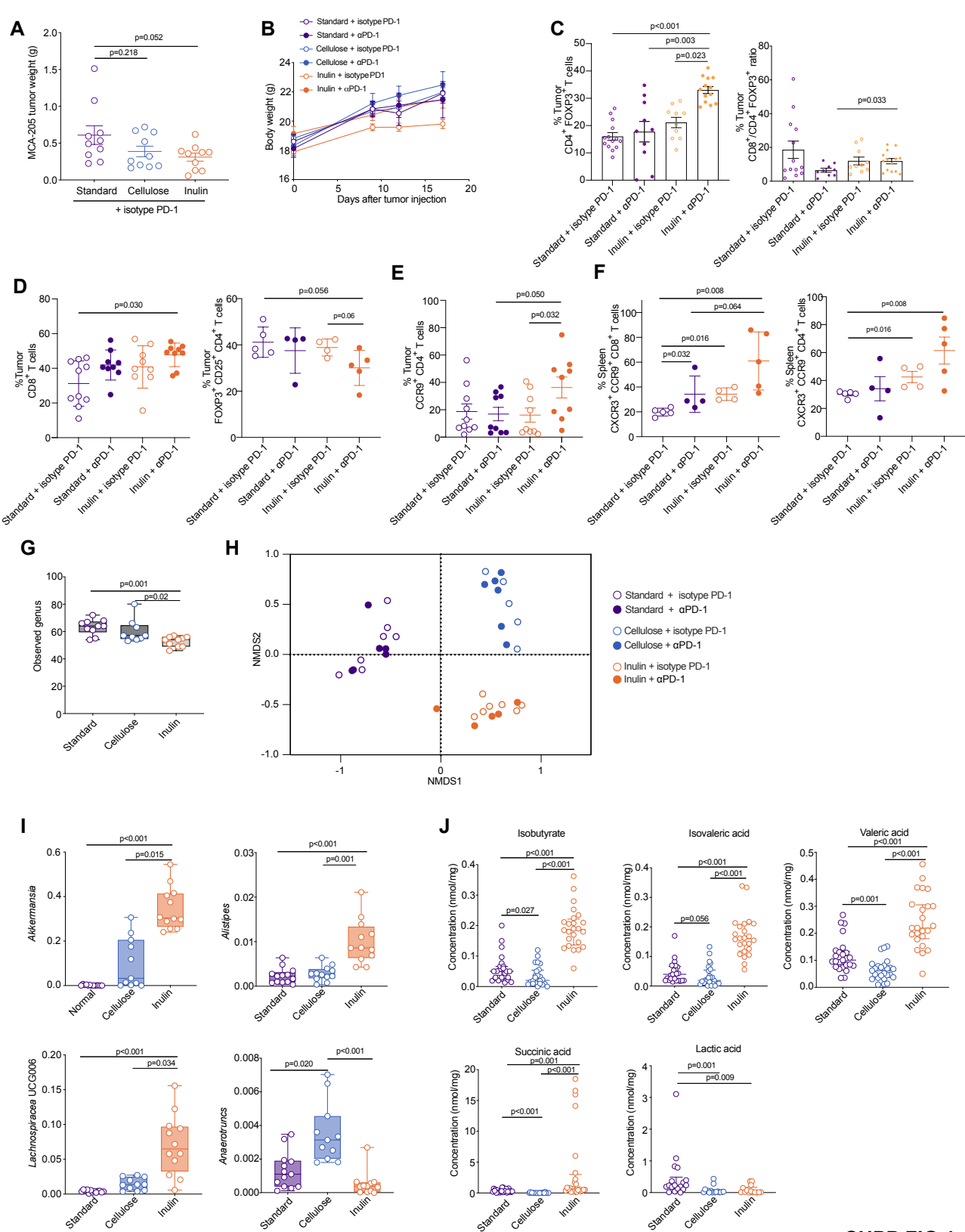

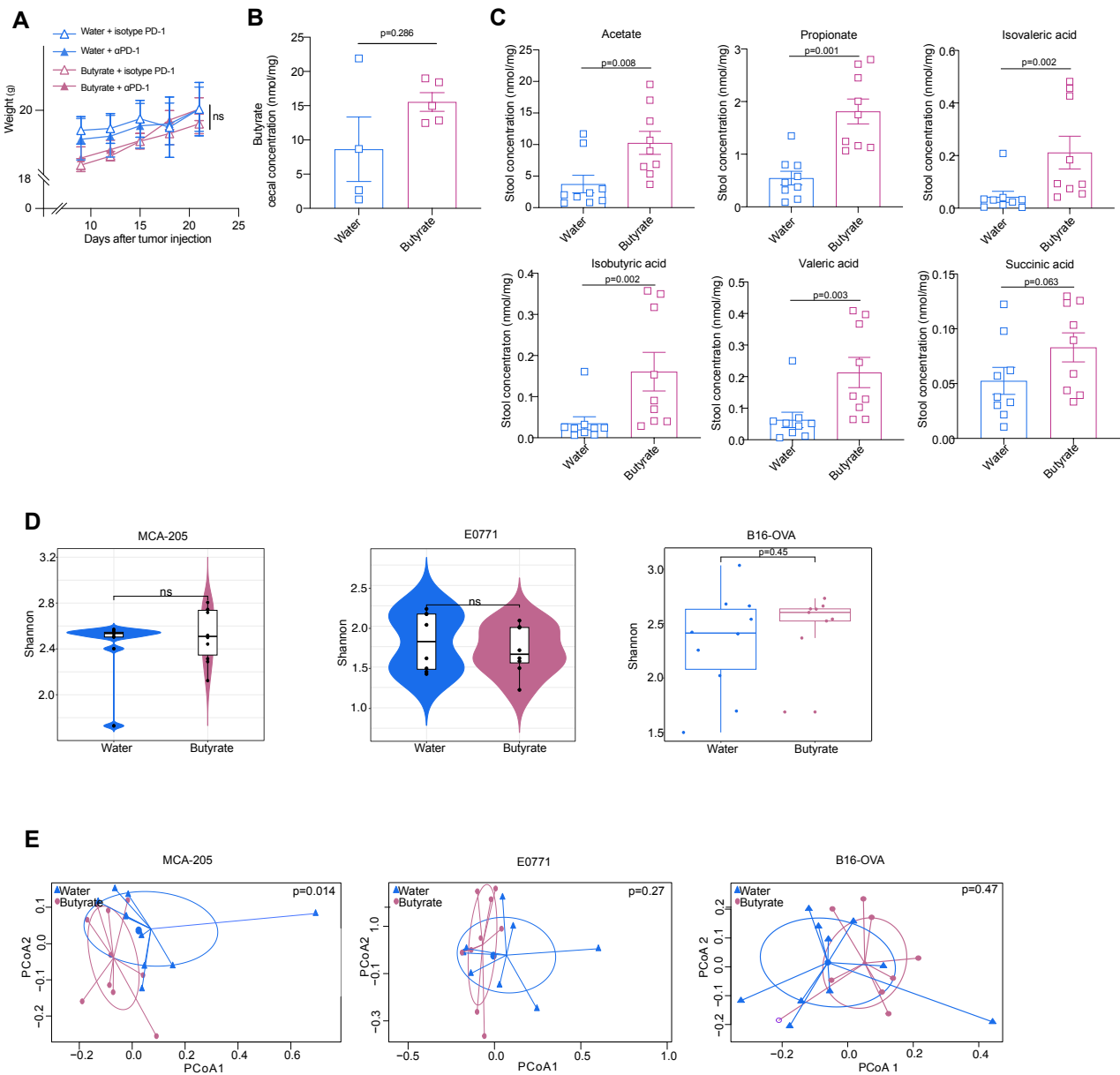



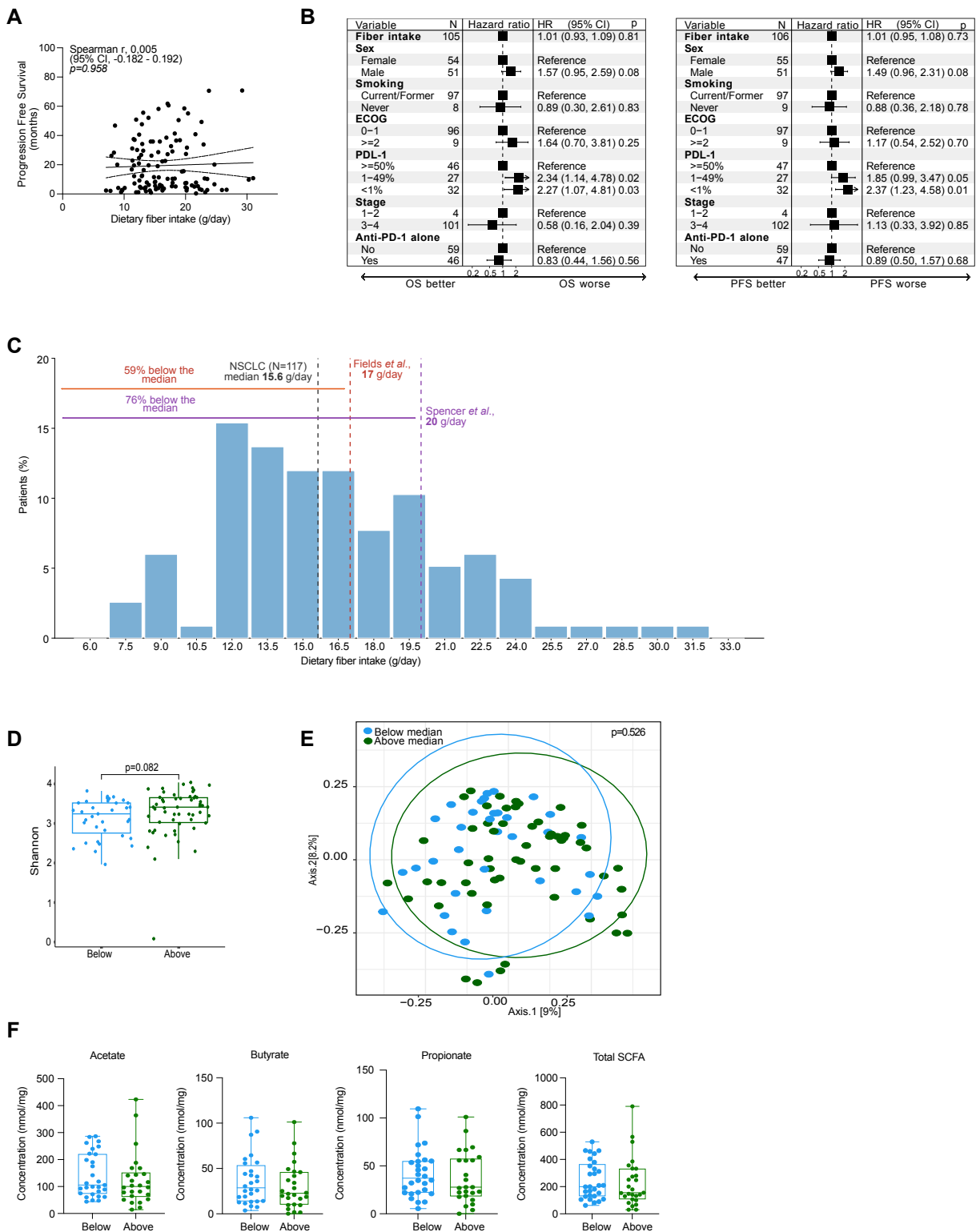

Supplement: Supplementary Material — Final_figures_02Apr [file KGMI_A_2699457_SM3168.pdf]
