# Supplementary material for: Dietary intervention through bacterial-derived butyrate elicits anti-tumor activity and increases anti-PD-1 response
Source: Gut Microbes. 2026 Jul 15;18(1):2699457. doi: 10.1080/19490976.2026.2699457 (PMC13374751; doi:10.1080/19490976.2026.2699457)
Supplement: Supplementary_captions.docx [file KGMI_A_2699457_SM3167.docx]

**Supplementary Figure 1. Inulin supplementation alters the gut microbiome composition and increases effector T cells frequency.**

**(A)** MCA-205 tumor weight at sacrifice of mice fed with a standard (n=10), cellulose (n=10) or inulin (n=10) diet and treated with an isotype control. Results are shown from combined data from two independent experiments; n refers to the number of mice analyzed per group. **(B)** Body weight kinetics of mice fed with a standard (n=5), cellulose (n=5) or inulin (n=5) diet and treated with an isotype control or 𝛂PD-1 therapy. **(C)** Multiplex analysis of tumor sections from standard and inulin diet (isotype or 𝛂PD-1) representing the frequency of FoxP3^-^ CD4^+^ T cells and the ratio of CD8^+^/FoxP3^+^ CD4^+^ T cells. Each dot represents one individual tumor. Data from different biological and technical replicates were included in the analysis. Flow cytometry analysis of CD8+ T cells and FoxP3^+^ CD25^+^ CD4^+^ T cells **(D)** and CCR9^+^ CD4^+^ T cells **(E)** expression in tumor of MCA-205 tumor-bearing mice treated with either standard or inulin diet (isotype or 𝛂PD-1). **(F)** Flow cytometry analysis of CXCR3^+^ CCR9^+^ CD8^+^ and CD4^+^ T cells expression from the spleen of MCA-205 tumor-bearing mice treated with either standard and inulin diet (isotype or 𝛂PD-1). **(G)** Alpha diversity of the standard, cellulose and inulin diet mice gut microbiome, assessed by 16S rRNA gene sequencing, represented by the observed genus. The box represents the interquartile range, the median line indicates the median, and the whiskers represent the extreme values**. (H)** Non-metric multidimensional scaling (NMDS) plot based on Bray-Curtis dissimilarities derived from log-transformed bacterial community composition (V3-V4 16S rRNA gene data) from MCA-205 experiment. **(I)** Boxplot representation of *Akkermansia*, *Alistipes*, *Lachnospiracea UCG.006* and *Anaerotruncus* relative abundance based on 16S rRNA gene sequencing from fecal samples collected from MCA-205 mice experiment (standard n=13, cellulose n=11, and inulin n=12). **(J)** Fecal concentrations of SCFAs in MCA-205 mice fed with the indicated diets: total SCFA (standard n = 193; cellulose n = 184; inulin n = 182), acetate (n = 25, 23, 24), butyrate (n = 25, 25, 24), and propionate (n = 25, 22, 24).

**Supplementary Figure 2. Butyrate supplementation to mice replicates anti-tumor responses observed with an inulin-rich diet.**

**(A)** Body weight kinetics of water control or butyrate mice in the presence of an isotype control or 𝛂PD-1 treatment (n= 5 mice/group). **(B)** Cecal concentration of butyrate from E0771 tumor bearing mice after receiving either water or butyrate. **(C)** Fecal concentration of SCFA from E0771 tumor bearing mice after receiving either water or butyrate. **(D)** Alpha diversity of water control and butyrate mice gut microbiome, assessed by 16S rRNA gene sequencing, represented by the Shannon index from MCA-205 (left), E0771 (middle) and B16-OVA (right) tumor model. **(E)** Principal coordinates analysis (PCoA) plot based on Bray–Curtis dissimilarities derived from 16S rRNA gene (V3–V4) community profiles from the MCA-205 (left), E0771 (middle), and B16-OVA (right) tumor models.

**Supplementary Figure 3. Butyrate impacts tumor and CD8^+^ T cells populations and transcriptomics profile.**

**(A)** Flow cytometry analysis of CCR9^+^ CXCR3^+^ CD4^+^ T cells and CCR9^+^ CXCR3^+^ CD8^+^ T cells expression from the spleen of B16-OVA tumor-bearing mice treated with either water or butyrate in the presence of isotype (respectively, n=8; n=9 mice) or 𝛂PD-1 treatment (respectively, n=9;n=10 mice). Results are shown from combined data from two independent experiments.Venn diagram and UpSet plot illustrating the overlap of downregulated differentially expressed genes (DEG) from bulk B16-OVA tumors **(B)** or CD8+ TILs **(C)** among indicated treatment comparisons (padj < 0.05 and FC > 0.8). Numbers represent unique or shared DEGs. **(D)** ImmuCellAI analysis of tumor-infiltrating immune populations showing normalized expression levels of CD8^+^ T cells, cytotoxic CD8^+^ T cells, and central memory CD8^+^ T cells in tumors from mice supplemented with water or butyrate and treated with either isotype control or anti–PD-1 antibody. **(E)** ImmuCellAI analysis of tumor-infiltrating immune populations showing normalized expression levels of CD4^+^ T cells, CD4^+^ T regulatory cells in tumors from mice supplemented with water or butyrate and treated with either isotype control or anti–PD-1 antibody. **(F)** Unsupervised hierarchical clustering of CD8⁺ TIL RNA-seq profiles showing treatment-associated differences in the expression of selected metabolic/AhR and trafficking/adhesion genes across groups (water or butyrate, ± anti–PD-1). (G) Normalized RNA-seq counts of *CXCL9* and *CXCL10* in bulk B16-OVA tumors from mice supplemented with water or butyrate and treated with either isotype control or anti–PD-1 antibody. Flow cytometry analysis of CCR9 frequency **(H)**, α4β7 frequency **(I) and** α4β7 MFI **(J)** on CD4⁺ and CD8⁺ T cells stimulated in vitro in the presence of butyrate (0.5 or 1mM) or Acetate (10mM). Flow cytometry analysis of CCR9 frequency and MFI **(K)** and α4β7 frequency and MFI **(L)** on CD4⁺ and CD8⁺ T cells stimulated in vitro in the presence of butyrate (1mM) or Acetate (5mM) and in addition to CH22 or FICZ treatment. Results are shown as mean ± SEM. Statistical significance was assessed using the Mann–Whitney U test or Friedman’s test, followed by Dunn’s multiple comparison test.

**Supplementary Figure 4. High fiber consumption is not associated with significant improved clinical responses or fecal microbial diversity indexes in NSCLC patients.**

**(A)** Scatter plot showing the correlation between total dietary fiber intake (g/day) and progression-free survival (PFS) in NSCLC patients (n=117).

**(B)** Multivariable Cox proportional hazards models for overall survival (OS) and PFS in the NSCLC cohort, including dietary fiber intake and clinical covariates. Forest plots show hazard ratios (HRs) with 95% confidence intervals (CIs) from multivariable Cox regression models including dietary fiber intake (continuous), sex, smoking status, ECOG performance status, PD-L1 expression category, stage, and anti–PD-1 regimen (anti–PD-1 alone vs other). HR > 1 indicates worse outcome and HR < 1 indicates better outcome. Analyses include patients with complete covariate information (OS: n = 105; PFS: n = 106). **(C)** Histogram showing the distribution of total dietary fiber intake (g/day) in NSCLC patients (n = 117). The dashed black line indicates the cohort median (15.6 g/day). Dashed red and purple lines indicate the thresholds at 17 g/day and 20 g/day respectively. Shotgun metagenomic analyses showing alpha diversity using Shannon index **(D)**, and beta diversity assessed using principal coordinates analysis (PCoA) based on Bray–Curtis dissimilarity **(E)**. **(F)** Fecal SCFA concentration (nmol/mg) in NSCLC, patients were segregated into below or above the dietary fiber intake median.

|  | Standard chow (Teklad 2018) | 10% Cellulose diet (TD.190723) | 10% Inulin diet (TD.190651) |
| --- | --- | --- | --- |
| Fiber source | Insoluble fiber, including cellulose, hemicellulose, and lignin | Cellulose | Inulin |
| Protein (%) | 18.6 | 17.7 | 17.7 |
| Fat (%) | 6.2 | 5.2 | 5.2 |
| Carbohydrate (%) | 44.2 | 60.5 | 65.5 |
| Fiber (%) | 14.7 | 10 | 10 |
| Energy density (kcal/g) | 3.1 | 3.6 | 3.8 |

**SupplementaryTable 1.** Fiber source and other macronutrient composition of the experimental diets.

| Baseline characteristics | NSCLC patients | |  |
| --- | --- | --- | --- |
|  | Below fiber median (n=58) | Above fiber median (n=59) |  |
| Age | 73 | 74 |  |
| Sex | | | p=0.097 |
| Female | 25 (21.4%) | 35 (29.9%) |  |
| Male | 33 (28,2%) | 24 (20.5%) |  |
| BMI | 23,3 | 25 |  |
| Smoking habit | | | p=0.001 |
| Current | 18 (15.4%) | 7 (6%) |  |
| Former | 40 (34.2%) | 43 (36.8%) |  |
| Never | 0 | 5 (4.3%) |  |
| NA | 0 | 4 (3.4%) |  |
| ECOG performance-status score | | | p=0.747 |
| 0 | 22 (18.8%) | 20 (17.1%) |  |
| 1 | 31 (26.5%) | 32 (27.4%) |  |
| 2 | 5 (4.3%) | 5 (4.3%) |  |
| 3 | 0 | 2 (1.7%) |  |
| Histology | | | p=0.446 |
| Adenocarcinoma | 49 (41.9%) | 51 (43.6%) |  |
| Squamous | 8 (6.8%) | 5 (4.3%) |  |
| Others NSCLC | 1 (0.9%) | 3 (1.7%) |  |
| PDL-1 IHC expression | | | p=0.935 |
| <1% | 14 (12%) | 17 (14.5%) |  |
| 1-49% | 15 (12.8%) | 14 (12%) |  |
| >50% | 26 (22.2%) | 26 (22.2%) |  |
| NA | 3 (2.6%) | 2 (1.7%) |  |
| Stage | | | p=0.344 |
| IIA-B | 2 (1.7%) | 2 (1.7%) |  |
| IIIA | 2 (1.7%) | 5 (4.3%) |  |
| IIIB | 3 (2.6%) | 7 (6%) |  |
| IV | 51 (43.6%) | 44 (37.6%) |  |
| IVA | 0 | 1 (0.9%) |  |
| Treatment | | | p=0.147 |
| Anti-PD-1 | 23 (19.7%) | 29 (24.8%) |  |
| Anti-PD-1 + Chemotherapy | 31 (26.5%) | 24 (20.5%) |  |
| Anti-PD-1 + anti-CTLA-4 | 1 (0.9%) | 0 |  |
| Others | 5 (4.3%) | 4 (3.4%) |  |
| RECIST | | | p=0.592 |
| PD | 10 (8.5%) | 13 (11.1%) |  |
| SD | 25 (21.4%) | 22 (18.8%) |  |
| PR | 17 (14.5%) | 21 (17.9%) |  |
| CR | 6 (5.1%) | 3 (2.6%) |  |

**Supplementary Table 2. Baseline clinical characteristics of NSCLC patients according to fiber intake status.**

Variables are expressed as median and percentages (%). Group comparisons were performed using Fisher’s exact test.

*Abbreviations*: BMI: body mass index; ECOG: Eastern Cooperative Oncology Group; IHC: Immunohistochemistry; PD-L1: Programmed Death-Ligand 1, PD: progressive disease; SD: stable disease; PR: partial response; CR: complete response; NA: not available.

**Supplementary Table 3. Baseline clinical characteristics of NSCLC patients selected as fecal donors for FMT experiments, stratified by fecal butyrate concentration (Low vs High).**

Variables are reported as mean (SD) or n (%), as indicated.

Abbreviations: ATB: antibiotics; BMI: body mass index; ECOG: Eastern Cooperative Oncology Group performance status; ICI: immune checkpoint inhibitor; PD-L1: Programmed Death-Ligand 1; PD: progressive disease; PR: partial response; CR: complete response; NA: not available.
